# Serum phospholipids and sphingolipids are linked to early-stage osteoarthritis by lipidomic profiling

**DOI:** 10.1186/s13075-025-03537-4

**Published:** 2025-03-31

**Authors:** Gerrit Eichner, Gerhard Liebisch, Christiane Hild, Markus Rickert, Juergen Steinmeyer

**Affiliations:** 1 Mathematical Institute, Justus Liebig University Giessen, Giessen, Germany; 2 Department of Clinical Chemistry and Laboratory Medicine, University Hospital Regensburg, Regensburg, Germany; 3 Laboratory for Experimental Orthopaedics, Department of Orthopaedics and Orthopaedic Surgery, Justus Liebig University Giessen, Giessen, Germany

**Keywords:** Phospholipids, Sphingolipids, Osteoarthritis, Serum, Synovial fluid, Lipidomic, Mass spectrometry, ESI-MS/MS, Biomarker, Systemic response

## Abstract

**Background:**

Osteoarthritis (OA) is associated with abnormal lipid metabolism, wherein elevated levels of phospholipids (PLs) and sphingolipids (SLs) in human and canine synovial fluid (SF) have been observed. The aim of this lipidomic study was to evaluate how closely blood lipid levels reflect changes in SF, building on previous findings.

**Methods:**

Lipids were extracted from knee SF and serum of 44 joint-healthy donors and 58 early (eOA) or late OA (lOA) patients. By electrospray ionization tandem mass spectrometry (ESI-MS/MS), we quantified the extracted lipids and conducted comprehensive statistical analyses.

**Results:**

Human SF and serum had similar PL and SL compositions. Quantifying 91 lipid species from 6 major classes revealed OA-related changes in serum, with the lowest levels in healthy controls and elevated levels already in the eOA cohort. Generally, serum PL and SL levels were 3–12 times higher than in SF. Specific PL species were elevated in both SF and serum of eOA and lOA patients compared to healthy controls, while nearly 10% of the PL species measured were higher exclusively in the serum of OA patients.

**Conclusions:**

The significant lipidomic alterations that were detected at an average Outerbridge score of less than 2 suggest that certain serum PLs may serve as indicators for monitoring the early stages of OA even before radiologic detection is possible. With 10% of PL species elevated only in OA serum, our data implicate the existence of a systemic response that parallels the local lipid metabolic response to OA.

**Supplementary Information:**

The online version contains supplementary material available at 10.1186/s13075-025-03537-4.

## Introduction

Osteoarthritis (OA) is the most prevalent chronic disorder of the entire joint and has a long and asymptomatic early phase [1, 2]. OA induces substantial pain, diminishes joint mobility, and ultimately leads to disability. Currently, OA is diagnosed only at an advanced stage of disease, based on clinical symptoms and radiological imaging. Consequently, the exploration of alternative early diagnostic methods has intensified.

Lipids are essential for cell membranes, energy stores, and regulation of signaling and metabolism, and this spectrum of functions is reflected in their myriad chemical structures. For instance, recent studies have shown that individual lipid species, such as the 3 chemically similar lysophosphatidylcholine (LPC) species, as well as sphingolipid (SL) species, can stimulate fibroblast-like synoviocytes (FLSs) of OA knee joints to synthesize proteins that influence inflammatory, anabolic, catabolic, and apoptotic mechanisms [3, 4]. Our prior lipidomic studies have demonstrated that the lipid profile in human knee synovial fluid (SF) is associated with joint health status [5, 6]. By quantitative mass spectrometric analysis of knee SF, we found that the concentrations of phospholipids (PLs), SLs, and minor lipid species often differed between healthy individuals, the early stage of OA (eOA) and late stage of OA (lOA), and rheumatoid arthritis (RA) [5, 6]. Our lipidomic analysis of knee SF using the canine “groove” model of OA [7] and a study by Pousinis et al. [8] of plasma in a mouse model of OA suggest that elevated levels of PLs and SLs in human SF are associated with progression of OA and pain, regardless of mammal species.

The endogenous lipidome of mammals is influenced by genes, environmental factors, and their interactions, wherein changes in lipid profiles are caused in particular by genetic variation, diet, de novo lipogenesis, and enzymatic activity [9, 10]. In a recent study, 9 differentially expressed genes and 2 hub genes that are associated with lipid metabolism in OA were identified by bioinformatic analysis and the use of machine learning algorithms [11]. In addition, 48 genes that are related to the SL metabolic pathway were recently reported to be significantly upregulated in the OA synovium, of which 4 hub genes that correspond to proinflammatory cytokines and immune-related cells were identified in peripheral blood [12].

The exact source of each lipid class and type in SF is only partially known or has been described more generally. PLs and SLs in SF originate in part from synovial blood vessels and local production. Cells of the SF border, such as articular cartilage, synovium, and meniscus, contribute to locally derived or secreted lipids [13–17]. Our previous findings have highlighted the impact of transforming growth factor beta 1 and insulin-like growth factor 1 in stimulating OA FLSs to synthesize increased amounts of PLs, whereas interleukin-1ß, via upregulation of cholesterol hydroxylases and ATP-binding cassette A1 transporters, induces their release [13, 17–19]. Notably, the synovial membrane of OA patients can be distinguished from healthy tissue-derived membrane by its lipidomic profile, and differential lipid levels have recently been demonstrated in mouse cartilage and bone [15, 20]. Lipids are stored in chondrocytes and the extracellular matrix of articular cartilage [21–23].

Most PLs and SLs in SF, predominantly phosphatidylcholine (PC), appear to originate via diffusion from fenestrated blood capillaries near the synovial surface, the pores of which primarily face the synovial cavity [24]. As a result, SF can be viewed as an ultrafiltrate of plasma that is supplemented with locally produced factors, such as cytokines, growth factors, and lubricants [25].

Blood is a highly dynamic compartment that interacts and communicates with every tissue and organ. Thus, blood metabolic components can reflect the current status of health or disease throughout the body and can indicate biological processes that occur within the articular joints [26]. Despite the dynamic and interactive nature of blood, the correlation between the concentrations of 168 metabolites in blood and SF in patients with knee OA is modest [26]. In addition to the local cellular contributions, changes in lymphatic vessels can impact the composition of SF. In histochemical studies, the articular lymphatic system, implicated in the removal of macromolecules from the synovial space to draining lymph nodes, exhibits decreased vessel number in OA, potentially affecting lymph clearance and increasing macromolecule levels [27–29].

Lipidomic analyses have demonstrated characteristically altered fatty acid (FA), PL, and SL levels in the blood of patients with various diseases, such as Alzheimer’s disease; schizophrenia; RA; musculoskeletal pain; cancer; cardiovascular disease; diabetes; systemic lupus erythematosus; and renal, liver, and respiratory disease [30–38]. Similarly, our prior studies have shown changes in the PL and SL profiles of human RA knee SF and human and canine OA knee SF [5–7]. Although the analysis of SF can reveal important information for research purposes, it is too invasive for use as a routine diagnostic modality.

Thus, our current study tested the hypothesis that PLs and SLs in venous blood are associated with the progression of OA and constitute biomarkers for eOA. The objective was to assess whether serum PL and SL profiles mirror the alterations that are observed in SF. Partially paired serum and SF samples were analyzed by mass spectrometry in human cohorts with healthy and eOA and lOA knee joints. Collectively, our study provides evidence that serum lipid profiles reflect the early systemic and local response of PL and SL metabolism to initial alterations to articular joints in OA, enabling the identification of potential biomarkers of even early pre-radiographically detectable stages of OA.

## Methods

### Study design and participants

We obtained SF and serum simultaneously from 58 OA patients, categorized as having eOA (*n* = 29) or lOA (*n* = 29), based on a macroscopic examination of 6 cartilage surfaces, including the patella, trochlea, medial and lateral femur, and tibia, using the Outerbridge (OU) classification score. This score [39] was applied during arthroscopy or knee replacement surgery to assess cartilage damage on a scale of 0–4: normal cartilage (0), softening and swelling (1), fissuring without reaching bone or exceeding 1.5 cm in diameter (2), fissures reaching subchondral bone in an area over 1.5 cm in diameter (3), and cartilage erosions down to the subchondral bone (4). The average OU score (X OU) for the entire knee joint was calculated by dividing the accumulated scores by 6. The eOA was defined as X OU of ≤ 2, and X OU of > 2 was considered lOA. Radiographic severity was further characterized using Kellgren-Lawrence (K/L) grades of 0–4.

The inclusion criteria comprised either sex, age < 85 years, body mass index (BMI) ≤ 40, and C-reactive protein (CRP) ≤ 5 mg/L. Samples were excluded from patients with (1) other joint disease, such as RA, gout, joint infection, or trauma; (2) surgery within the last 3 months or knee joint surgery within the 6 months prior to study onset; (3) severe disease, such as HIV infection, tumor near the joint, diabetes mellitus, severe liver or kidney disease, and drug abuse; and (4) consumption of immunosuppressive drugs, lipid-lowering drugs, corticosteroids, or intra-articular hyaluronate during the preceding 3 months. Before serum and SF were obtained, we assessed the scanned laboratory and medical reports to record the medical history, medications taken, and concomitant diseases.

SF was collected from the knee joints of 13 postmortem donors without documented joint disease, providing a valid comparison with serum values. Serum was also obtained from 31 young volunteers without joint or other diseases, serving as a healthy control group, given their young age. Key demographics and clinical characteristics of the SF and serum donors are summarized in Table 1. The participants in this study were prospectively and consecutively enrolled in the Department of Orthopaedics and Orthopaedic Surgery, University Hospital Giessen and Marburg (UKGM, Giessen, Germany).

**Table 1:** Demographic and clinical characteristics of patients

	Healthy controls (serum) *n* = 31	Postmortem donors (SF) *n* = 13	Patients early OA (serum + SF) *n* = 29	Patients late OA (serum + SF) *n* = 29
Age [years]	26 (24–32) *n* = 31	24 (21–28) *n* = 13	41 (33–54)*** *n* = 29	67 (57–71)*** *n* = 29
female/male	12/19	1/12	13/16	13/16
BMI	23.1 (22.2–24.7) *n* = 31	24.8 (21.4–25.2) *n* = 13	26.4 (24.0-29.4) *n* = 29**	29.3 (26.2–31.4) *n* = 29***
CRP	0.5 (0.5–0.6) *n* = 31	NA	0.8 (0.5–1.3) *n* = 29	1.5 (0.8–2.1)** *n* = 29
OU-score	NA	NA	1.1 (0.7–1.5) *n* = 29	3.2 (2.7–3.5) *n* = 27
K/L-score	NA	NA	1.0 (0.0–1.0) *n* = 22	3.0 (3.0–4.0) *n* = 26
Cholesterol	178 (151–206) *n* = 29	NA	201 (177–222)* *n* = 28	223 (198–259)*** *n* = 28
HDL Chol.	58 (46–64) *n* = 29	NA	51 (40–57) *n* = 28	58 (46–67) *n* = 25
LDL Chol.	102 (87–128) *n* = 29	NA	124 (98–141)* *n* = 28	122 (109–156)*** *n* = 25
VLDL Chol.	20 (15–25) *n* = 29	NA	29 (23–32)* *n* = 28	31 (24–42)*** *n* = 25
TG	91 (79–134) *n* = 29	NA	133 (102–167)* *n* = 28	149 (131–176)*** *n* = 28
Apo-AI	150 (131–166) *n* = 26	NA	161 (143–173) *n* = 28	164 (152–193)* *n* = 19
Apo B-100	90 (75–100) *n* = 26	NA	100 (85–120)* *n* = 28	114 (95–128)*** *n* = 19

Data are presented as medians with interquartile range in brackets, and the concentrations of parameters were determined in serum. BMI, body mass index [kg/m^2^]; Apo-AI, apolipoprotein AI [mg/dL]; Apo B-100, apolipoprotein B-100 [mg/dL]; CRP, C-reactive protein [mg/L]; HDL Chol, High Density Lipoprotein Cholesterol [mg/dL]; K/L-score, Kellgren Lawrence score; LDL Chol, Low density Lipoprotein Cholesterol [mg/dL]; OU-score, Outerbridge score; SF, synovial fluid; TG, triglyceride [mg/dL]; VLDL Chol, Very Low Density Lipoprotein Cholesterol [mg/dL]; NA, not available. Statistically significant differences versus controls were determined using Welch´s two sample t-test after log-transformation and are indicated by p-values: * 0.05 > *p* > 0.01, ** 0.01 ≥ *p* > 0.001, *** 0.001 *≥* p

### Sampling of synovial fluid

SF was collected within several hours to 3 days postmortem during autopsies at the Institute of Forensic Medicine, University of Giessen (Germany). The correlation analysis of the SF values of the PL and SL classes and some exemplary species per lipid class with the postmortem time showed that the PL and SL values remained stable [5]. SF was also obtained during arthroscopy (eOA) or knee prosthesis implantation (lOA) and was processed immediately, as published [5, 6]. Briefly, samples were characterized for color, turbidity, and blood contamination, with turbid or blood-contaminated samples discarded. After incubation for 15 min at 37 °C, SF was passed through a 1.2-µm filter and analyzed by light microscopy to verify that they were free of cells and cellular debris. Subsequently, 10% (v/v) protease and phospholipase inhibitors were added, and samples were centrifuged (16,100 x g, 45 min, room temperature) to remove cellular particles. The supernatant was frozen at -86 °C for subsequent analysis. Some SF samples were reused from prior publications [5, 40] to increase the sample size.

### Sampling of human serum

Nonfasted blood samples were collected via peripheral venipuncture into 7.5-mL serum tubes with clot activator. After clotting at room temperature, the tubes were centrifuged at 590 x *g* for 10 min at 4 °C. Serum was harvested, aliquoted, and stored at -86 °C for further analysis.

### Extraction and mass spectrometry of lipids

Fine chemicals were sourced from Sigma (Taufkirchen, Germany) unless stated otherwise. HPLC-grade methanol and chloroform, gentamycin sulfate, and neomycin sulfate were obtained from Merck (Darmstadt, Germany). Lipids were extracted from cell-free and cellular debris-free SF and serum samples using the Bligh and Dyer method [41]. The extraction involved the use of non-naturally occurring lipid species as internal standards, provided by Avanti Polar Lipids (Alabaster, AL, USA) [5, 42, 43]. The concentrations of PL and SL species were measured by electrospray ionization tandem mass spectrometry (ESI-MS/MS) on a Quattro Ultima™ triple quadrupole mass spectrometer (Micromass, Manchester, United Kingdom). The analytical setup, correction of isotopic overlap of lipid species, and data analysis were performed for each lipid class as described [43]. A precursor ion scan of mass/charge (*m/z*) 184, specific for phosphocholine-containing lipids, was used for PC, sphingomyelin (SM), and LPC. A neutral loss of 141 and 185 was used for phosphatidylethanolamine (PE) and phosphatidylserine [44], respectively. Phosphatidylethanolamine-based plasmalogens (PE P) and phosphatidylglycerol were analyzed as described [44]. Briefly, fragment ions at m/z 364, 380, and 382 were used for PE P-16:0, PE P-18:1, and PE P-18:0 species, respectively. Sphingosine-based ceramides (Cer) were analyzed using a fragment ion of m/z 264 [45]. Correction of isotopic overlap of lipid species and data analysis were performed using self-programmed Excel macros for all lipid classes as described [43].

PL and SL species were annotated according to the shorthand notation of lipid structures analyzed by mass spectrometry [46]. Glycerophospholipid species annotation was based on the assumption of even numbered carbon chains only. The annotation of SM species is based on the assumption that a sphingoid base with two hydroxyl groups is present. Only lipid species with median concentrations higher than 1% of the median total of the corresponding class were evaluated further.

### Analysis of cholestesol, triglycerides, and apolipoproteins

Cholesterol, high-density lipoprotein (HDL) cholesterol, low-density lipoprotein (LDL) cholesterol, very-low-density lipoprotein (VLDL) cholesterol, triglycerides, and apolipoproteins (Apo) Apo-AI and Apo B-100 were quantified at the Institute for Clinical Chemistry and Laboratory Medicine (University Hospital Regensburg, Germany) using routine analytical methods.

### Statistical analysis

Serum and SF lipid level distributions between cohorts were compared by Wilcoxon’s rank sum test and, for ratios of serum to SF lipid levels, using a custom-made ratio test that was based on the δ-method. To account for multiple testing, p-values were FDR-adjusted in suitable test families (Supplementary Table S1 + S2).

To assess the potential effects of OA severity on total and individual PL and SL levels, we performed log-linear regression analyses using the X OU as the predictor. Model assumptions were confirmed by conventional residual diagnostics, and regression p-values were FDR-adjusted. Although large cohort studies have reported an association of age, sex, and BMI with the plasma lipidome [47, 48], our regression analyses were not adjusted for them for several reasons. PL and SL levels in serum showed no significant Pearson correlation with age or BMI across a wide range of ages and BMIs (Figure S1). Further, age has not been established to be a confounder, given that there is no evidence that age influences PL or SL synthesis in healthy joints. Including age and BMI as potential confounders in our regression models would have introduced unfavorable multicolinearity due to their correlation. We also excluded sex as a confounder, because there were no significant differences in log-transformed PL and SL levels between sexes by Student’s t-test (Figure S2).

The dependence of serum ratios between select LPC and PC species was also analyzed by simple linear regression. Model diagnostics were satisfactory, and multiple R² was used to quantify the explained variance in the response variables.

To determine whether the profiles of the PL and SL classes in serum paralleled those in SF, we used a log-linear mixed-effects ANOVA to analyze whether the differences between the mean log-transformed concentrations of all PL and SL classes in serum and SF were the same and thus constant.

To assess a possible effect of OA severity, increasing age, BMI, and sex on the combined 6-dimensional distribution of total PLs and SLs, cholesterol, LDL cholesterol, triglyceride, and Apo-AI, as well as Apo B-100 as a “general response”, a multivariate multiple linear regression model was fitted to the log-transformed response variables.

All statistical analyses and data presentations were conducted in R (version 4.3.3) [49].

## Results

### Donor characteristics

Following the collection of knee SF and serum samples from all OA patients, we measured the levels of PLs and SLs simultaneously. Serum was obtained from young healthy volunteers (*n* = 31), whereas SF was obtained from similarly aged postmortem donors (*n* = 13), which served as healthy adult controls with no reported history of knee joint disease (Table 1). Based on our exclusion criteria and the patient characteristics, no subjects had any confounding factors, such as brain diseases (Alzheimer’s disease, schizophrenia); RA; cancer; diabetes; systemic lupus erythematosus; and liver, renal, or respiratory disease [5, 30–35].

Some patients had comorbidities, including metabolic syndrome (eOA: *n* = 2; lOA: *n* = 4), coronary heart disease (lOA: *n* = 1), angina pectoris (lOA: *n* = 1), cardiac insufficiency (lOA: *n* = 1), hyperthyroidism (eOA: *n* = 1; lOA: *n* = 2), hypothyroidism (eOA: *n* = 1; lOA: *n* = 3), hay fever (eOA: *n* = 5; lOA: *n* = 1), osteoporosis (eOA: *n* = 1), spinal stenosis (lOA: *n* = 1), and scoliosis (lOA: *n* = 1).

### Distribution of lipids in human serum and synovial fluid

**Fig. 1:**
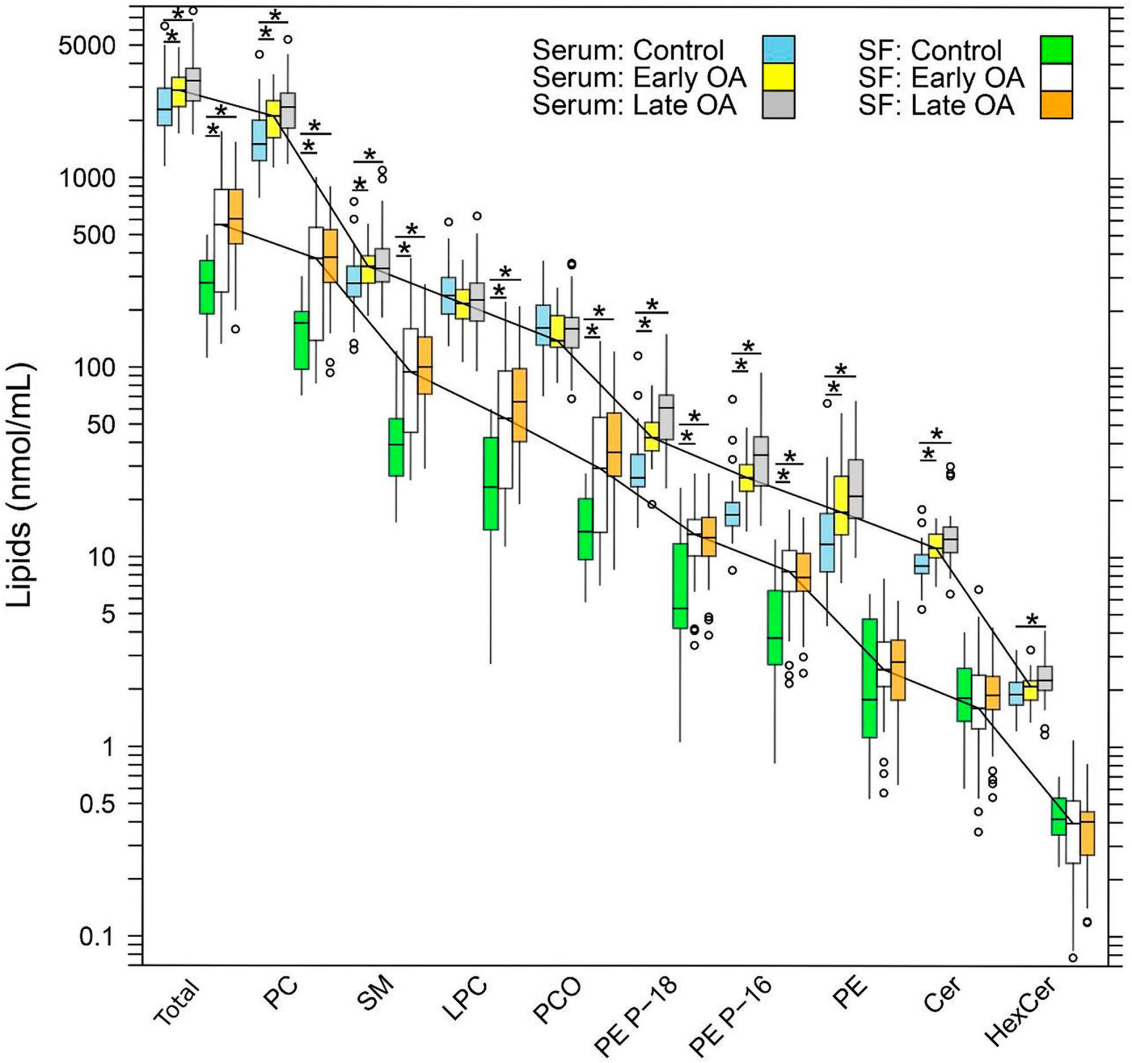
Lipid distribution in human serum and synovial fluid. Total phospholipid (PL) and sphingolipid (SL) content and the levels of PL and SL classes in synovial fluid (SF) and serum are depicted for 13 SF and 31 serum controls, 29 patients with early OA (eOA), and 29 patients with late OA (lOA). PL and SL species were quantified by ESI-MS/MS, and data are presented as box plots with median, interquartile range, and outliers. The solid polylines connect eOA group medians, illustrating elevated levels in serum compared with the corresponding SF levels. Statistically significant differences were determined by Wilcoxon´s rank sum test and are indicated by * if their FDR-adjusted p-values are < 0.05. Precise PL and SL concentrations, serum/SF ratios, and statistical results are listed in Supplementary Table S1. PC, phosphatidylcholine; PC O, ether-linked phosphatidylcholine; LPC, lysophosphatidylcholine; SM, sphingomyelin; PE, phosphatidylethanolamine; PE P, phosphatidylethanolamine-based plasmalogen; HexCer, hexosylceramide; Cer, ceramide

The human SF and serum samples had similar compositions of lipid classes and species, comprising 91 lipid species across 6 classes (Fig. 1, Supplementary Table S1 + S2): 28 PC containing 16 ether-linked phosphatidylcholine (PC O); 5 LPC; 13 SM; 24 PE P; 12 PE; and 9 Cer containing 2 hexosylceramide (HexCer) species. Whereas the profiles of lipid classes and species were similar in serum and SF, nearly all concentrations in SF were notably lower. Although PL and SL concentrations were occasionally higher in the lOA cohort versus the eOA cohort, the differences were marginal (Fig. 1). For instance, compared with control serum [2293 (1881–2964) nmoles/mL], the median total PL and SL content increased 1.26-fold in serum from patients with eOA [2899 (2369–3378) nmoles/mL; FDR-adj. *p* = 0.022] and 1.41-fold in serum from those with lOA [3254 (2537–3772) nmoles/mL; FDR-adj *p* = 0.002] (Supplementary Table S1). With similar profiles but much lower compared with the control cohort [278 (191–365) nmoles/mL], the median total PL and SL levels rose 2.0-fold [565 (249–868) nmoles/mL; FDR-adj. *p* = 0.014] and 2.2-fold [606 (447–866) nmoles/mL; FDR-adj. *p* < 0.001] in SF from patients with eOA and lOA, respectively (Supplementary Table S1).

By log-linear regression, total PL levels correlated significantly with OA disease stage in serum [ß = 0.04 (0.017; 0.067), *p* = 0.001], implying a rise in the mean total PL content of 9.6% (3.9%; 16.7%) per unit X OU, and in SF [ß = 0.07 (0.01; 0.13), *p* = 0.022], indicating a respective rise in the mean total PL level of 17.4% (2.3%; 34.9%). Based on the multiple R^2^ values, 12% and 8% of the measured variance in log-transformed total PL and SL levels in serum and SF, respectively, were related to the stage of OA.

### Relative distribution of phospholipid and sphingolipid classes

The distribution of lipid classes in serum and SF was similar between cohorts. For instance, PC was the predominant PL class in controls, constituting a median of 65.5% and 61.3% of all PLs and SLs in serum and SF, respectively. Other classes, such as SM (12.1% and 14.0%, respectively), LPC (10.4% and 8.4%, respectively), and PC O (7.1% and 4.9%, respectively), followed at lower proportions. The lipid classes PE, PE P, and Cer were present at lower concentrations, representing 0.4–1.9% of total PLs and SLs.

### Testing of parallelism between PL and SL profiles in serum and SF

To determine whether the distance between the averages of the log-transformed concentrations of the lipid classes in serum and SF was the same and thus the mean profiles parallel, we performed mixed-effects ANOVA. The low p-values (< 0.001) for the eOA and lOA cohorts indicated a significant interaction between the source (serum vs. SF) and lipid class, suggesting nonparallel profiles. Thus, the log-transformed lipid concentrations in Fig. 1 reflect median serum and SF profiles that only appear to be parallel.

Thus, we calculated the ratios of median concentrations of lipid classes in serum to those in SF, as well as the ratios of the 1st and of the 3rd quartiles (Supplementary Table S1). We found that the median concentrations of lipid classes and species were approximately 3–12 times higher in serum than in SF. Significant differences in the distribution of PL and SL classes between serum and SF were observed in OA patients compared with the control cohort (Supplementary Table S1). For instance, versus SF, the median total level of serum PLs and SLs was 8.2-fold higher in the control group versus 5.1-fold (compared with control: FDR-adj. *p* = 0.006) in eOA patients and 5.4-fold (compared with control: FDR-adj. *p* = 0.001) in the lOA cohort. Thus, our data show a higher serum-to-SF ratio for total PL and SL content in healthy controls versus OA patients. With regard to PL classes, this pattern was particularly evident for the PC, LPC, PC O, and SM classes, in contrast to Cer, PE, and PE P S1(Supplementary Table S1).

### Phospholipid and sphingolipid classes and species in serum and SF

**Fig. 2:**
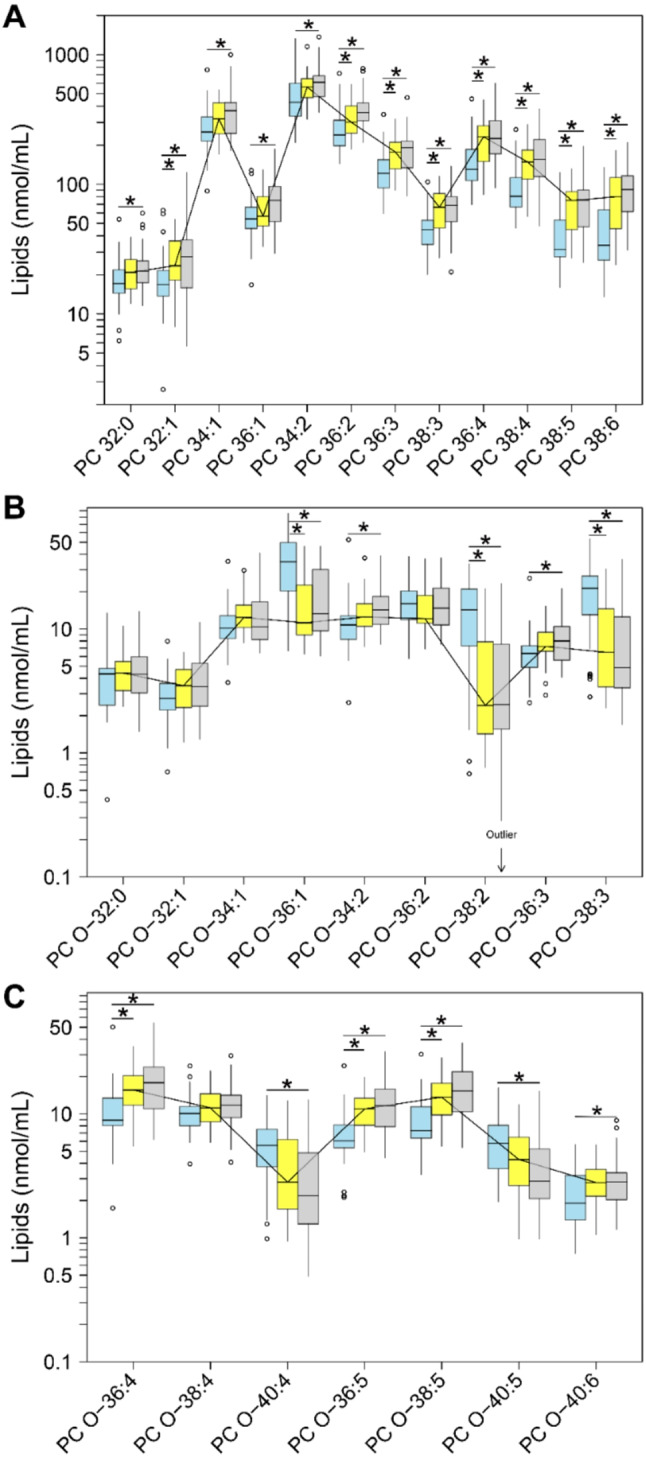
Concentrations of (**A**) phosphatidylcholine (PC) species and (**B**,** C**) ether phosphatidylcholine (PC O) species in serum. ESI-MS/MS quantification of serum samples from controls (*n* = 31, blue) and early stage OA (eOA, *n* = 29, yellow) and late stage OA (lOA, *n* = 29, grey) patients. Ether species were assigned based on the assumption that the PCs contained only fatty acids with an even number of carbon atoms. Data are presented as box plots with median, interquartile range, and outliers. The solid polylines connect the medians to support the visual impression. Statistically significant differences were determined by Wilcoxon´s rank sum test and are indicated by the FDR-adjusted p-values: * < 0.05. Precise concentrations, serum/SF ratios, and statistical results are listed in Supplementary Table S2

In serum and SF, we quantified 28 PC species that contained 16 PC O species with varying FA chain lengths and number of double bonds (Fig. 2, Supplementary Table S2). The PCs existed predominantly in unsaturated form—11 of the 12 PC species and 15 of the 16 PC O species contained at least 1 unsaturated FA. Compared with control serum [PC: 1503 (1228–2011) nmoles/mL; PC O: 162 (131–213) nmoles/mL], the median total PC and PC O content increased 1.4-fold (FDR-adj. *p* = 0.006) and declined 0.8-fold (FDR-adj. *p* = 0.245), respectively, in serum from patients with eOA and rose 1.6-fold (FDR-adj. *p* < 0.001) but was unchanged (FDR-adj. *p* = 0.66), respectively, in serum from lOA patients (Supplementary Table S1).

With similar profiles but much lower levels compared with the median levels of PC [171 (98–197) nmol/mL] and PC O [14 (10–20) nmoles/mL] in SF of the control cohort, these classes increased 2.2-fold (FDR-adj. *p* = 0.014) and 2.2-fold (FDR-adj. *p* = 0.017), respectively, in SF from patients with eOA and 2.2-fold (FDR-adj. *p* < 0.001) and 2.6-fold (FDR-adj. *p* < 0.001), respectively, in SF from lOA patients (Supplementary Table S1).

By log-linear regression of PC and PC O class levels and OA disease stage, we observed a significant association in serum only for PC [ß = 0.05 (0.02; 0.08), *p* < 0.001] and in SF for PC [ß = 0.07 (0.01; 0.13), *p* = 0.02] and PC O [ß = 0.09 (0.03; 0.16), *p* = 0.005]. These values imply a rise in the mean serum level of PC of 12.2% (4.7%; 20.3%) and in the mean SF level of PC of 17.5% (2.3%; 34.9%) and of PC O of 23.0% (7.1%; 44.6%) per unit X OU. Our log-linear regression analysis revealed a significant correlation between all 12 PC species and 12 of 16 PC O species in serum with OA disease stage, with FDR-adjusted p-values of less than 10%. However, in SF, a significant association was seen with OA progression for only 6 of 12 PC and 2 of 16 PC O species.

**Fig. 3:**
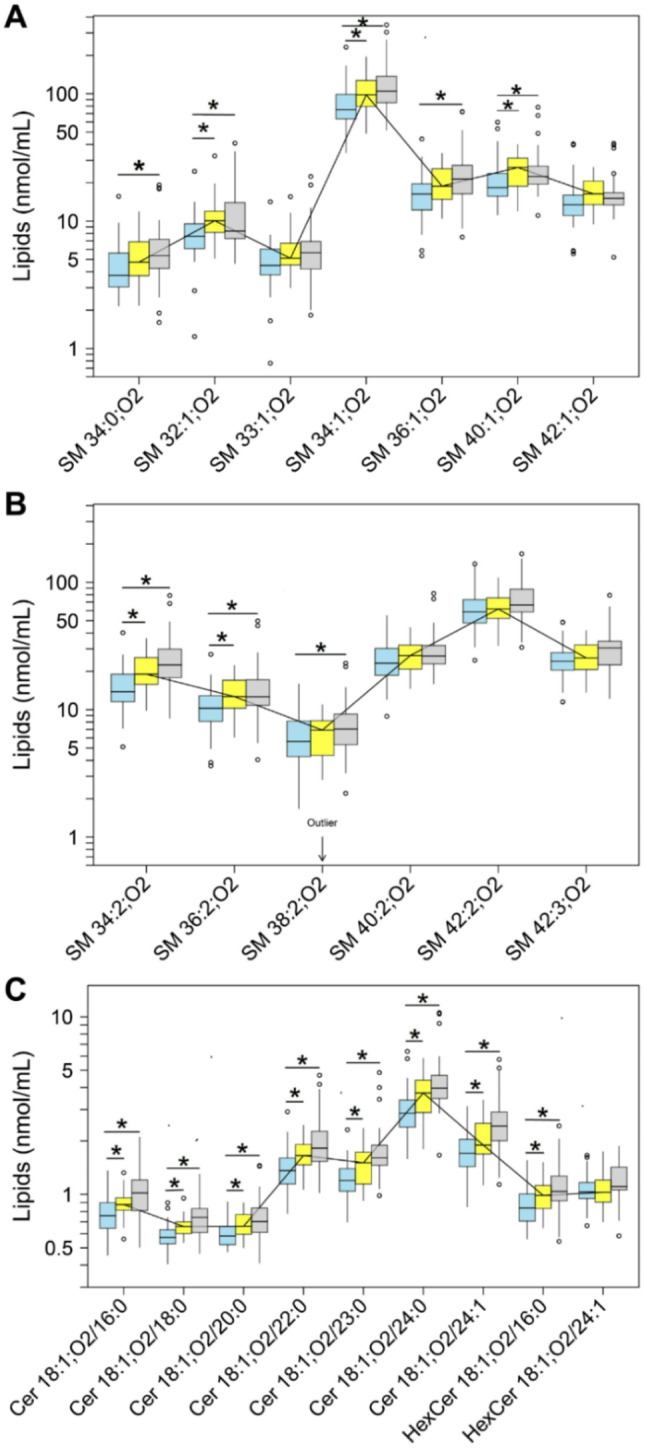
Concentrations of (**A-B**) sphingomyelin (SM) species and (**C**) ceramide (Cer, HexCer) species in serum. ESI-MS/MS quantification in serum samples from controls (*n* = 31, blue) and early stage OA (eOA, *n* = 29, yellow) and late stage OA (lOA, *n* = 29, grey) patients. The annotation of SM species is based on the principle that 2 hydroxyl groups are attached to a sphingoid base. For more information, refer to the caption of Fig. 2. Cer, ceramide; HexCer, hexosylceramide

Thirteen SM species with varying FA chain lengths and number of double bonds were quantified in serum and SF (Fig. 3, Supplementary Table S2). SM 34:1;O2 and SM 42:2;O2 were the most abundant SM species, accounting for 48% and 60% of total SM species in the serum and SF of the control cohort, respectively. Compared with control serum [278 (235–341) nmoles/mL], the median total SM content was 1.2-fold higher both in serum from patients with eOA (FDR-adj. *p* = 0.038) and lOA (FDR-adj. *p* = 0.009). Similarly, compared with the median level of SM in SF of the control cohort [39 (27–54) nmoles/mL], this content increased by 2.4-fold (FDR-adj. *p* = 0.017) in SF from patients with eOA, and 2.6-fold (FDR-adj. *p* = 0.001) in SF from those with lOA (Supplementary Table S1).

By log-linear regression of SM class levels and OA disease stage, there was a significant association in serum (ß = 0.04 (0.015; 0.07), *p* = 0.002), implying a rise in mean SM class levels of 9.6% (3.5%; 17.5%) per unit X OU, and in SF [ß = 0.07 (0.007; 0.13), *p* = 0.03], reflecting an increase in mean SM class levels of 17.5% (1.6%; 34.9%). In serum, all 13 SM species but only 6 of 13 SM species in SF correlated significantly with progression of OA, at an FDR of 10%.

**Fig. 4:**
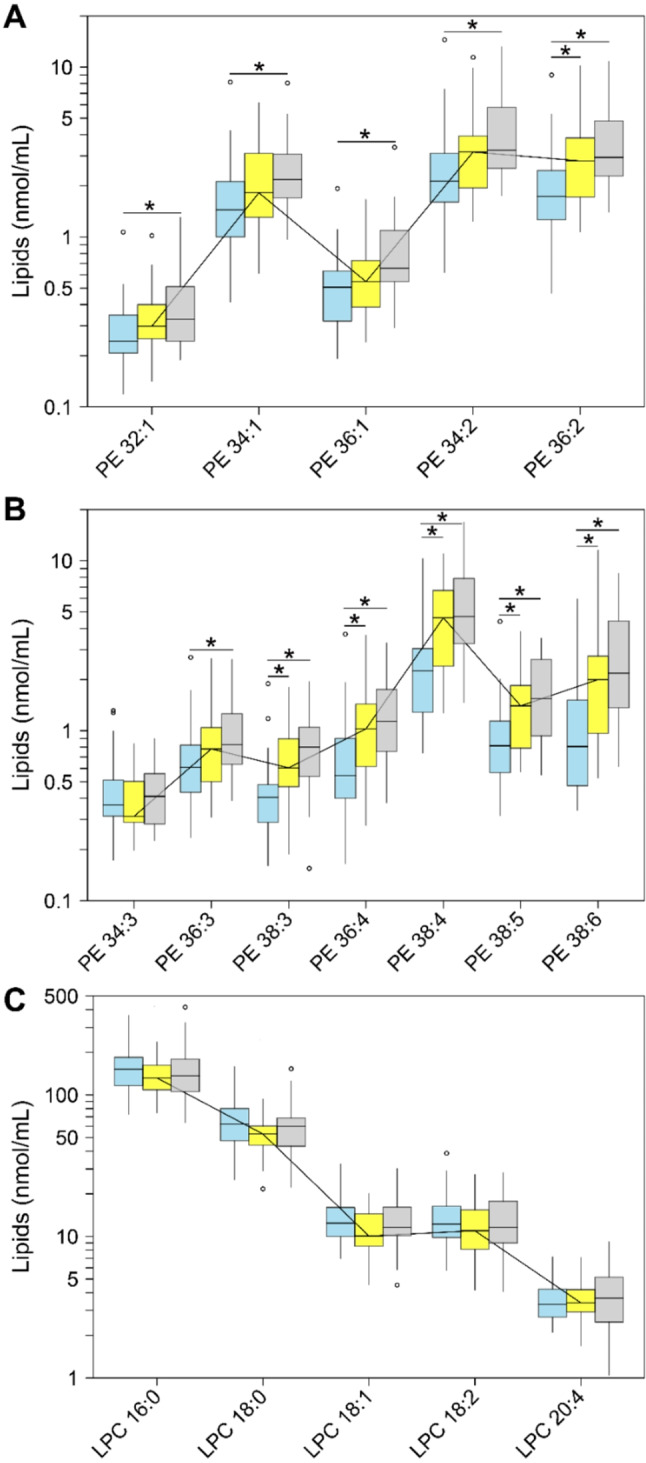
Concentrations of (**A-B**) phosphatidylethanolamine (PE) species and (**C**) lysophosphatidylcholine (LPC) species in serum. ESI-MS/MS quantification in serum samples from controls (*n* = 31, blue) and early stage OA (eOA, *n* = 29, yellow) and late stage OA (lOA, *n* = 29, grey) patients. Ether species were assigned based on the assumption that the PEs contain only fatty acids with an even number of carbon atoms. For more information, refer to the caption of Fig. 2

We quantified 5 LPC species with varying FA chain lengths and number of double bonds in serum and SF (Fig. 4, Supplementary Table S2). The most predominant LPC species was LPC 16:0, accounting for 63% and 47% of the LPC class in the serum and SF of the control cohort, respectively. Based on the number of carbon atoms, LPC species with 18 carbon atoms (18:0, 18:1, 18:2) constituted 36% and 47% of the LPC class in serum and SF of the control cohort, respectively. Compared with control serum [LPC: 240 (191–298) nmoles/mL], the median total LPC content was slightly lower by 0.9-fold both in serum from patients with eOA (FDR-adj. *p* = 0.111) and lOA (FDR-adj. *p* = 0.504). In contrast, relative to the median level of LPC in the SF of the control cohort [23 (14–43) nmoles/mL], this content was 2.3-fold higher (FDR-adj. *p* = 0.014) in patients with eOA and 2.8-fold higher (FDR-adj. *p* < 0.001) in those with lOA (Supplementary Table S1).

By log-linear regression of LPC class levels and OA disease stage, we noted a significant association only in SF [ß = 0.09 (0.018; 0.16), *p* = 0.015], implying a rise in mean LPC class level of 23.0% (4.2%; 44.5%) per unit X OU. Neither LPC class nor any of the 5 overall LPC species correlated significantly with OA disease stage in serum, at an FDR significance level of 10%. However, in SF, 2 of 5 LPC species were significantly linked to OA disease stage, at an FDR of 10%.

Twelve PE species with varying FA chain lengths and number of double bonds were quantified in serum and SF (Fig. 4, Supplementary Table S2). All PE species had unsaturated FAs, with 9 of the 12 PE species bearing FAs that had at least 2 double bonds. Compared with control serum [PE: 12 (8–17) nmoles/mL], the median total PE content was higher by 1.5-fold (FDR-adj. *p* = 0.015) in patients with eOA and by 1.8-fold (FDR-adj. *p* < 0.001) in those with lOA. Similarly, compared with the median level of PE in SF of the control cohort [2 (1–5) nmoles/mL], this value was higher by 1.4-fold (FDR-adj. *p* = 0.808) in SF from patients with eOA and by 1.6-fold (FDR-adj. *p* = 0.763) in those with lOA (Supplementary Table S1).

By log-linear regression analysis, serum PE class [ß = 0.08 (0.04; 0.12), *p* < 0.001] and 11 of the total 12 PE species were significantly associated with OA disease stage, at an FDR of 10%. Thus, our data imply a rise in mean PE class serum levels of 20.2% (9.6%; 31.8%) per unit X OU. However, no significant relationship was found between PE class or any PE species and OA progression in SF, at an FDR of 10%.

Nine Cer species, including 2 HexCer species, with varying FA chain lengths and number of double bonds, were quantified in serum and SF (Fig. 3, Supplementary Table S2). Of the 7 Cer species (excluding the 2 HexCer species), Cer 18:1;O2/24:0 and Cer 18:1;O2/24:1 collectively accounted for 42% and 41% of the total Cer and HexCer species in the serum and SF of the control cohort, respectively. Only 2 of the 9 Cer/HexCer species—Cer 18:1;O2/24:1 and HexCer 18:1;O2/24:1—had 1 unsaturated FA. Compared with control serum [Cer: 9 (8.2–10.4) nmoles/mL; HexCer: 1.9 (1.7–2.2) nmoles/mL], the median total Cer and HexCer content was higher by 1.2-fold (FDR-adj. *p* = 0.013) and 1.1-fold (FDR-adj. *p* = 0.245), respectively, in serum from patients with eOA and 1.4-fold (FDR-adj. *p* < 0.001) and 1.2-fold (FDR-adj. *p* = 0.009) in those with lOA. However, the median level of Cer [1.8 (1.4–2.6) nmoles/mL] and HexCer [0.4 (0.4–0.5) nmoles/mL] in the SF of the control cohort did not change significantly: 0.88-fold (FDR-adj. *p* = 0.893) and 0.95-fold (FDR-adj. *p* = 0.524), respectively, in patients with eOA and 1.0-fold (FDR-adj. *p* = 0.83) and 0.95-fold (FDR-adj. *p* = 0.382), respectively, in those with lOA (Supplementary Table S1).

A log-linear regression analysis detected a significant association for serum Cer [ß = 0.04 (0.023; 0.065), *p* < 0.001] and HexCer class [ß = 0.02 (0.004; 0.039), *p* = 0.016], wherein all 7 Cer species and 1 of 2 HexCer species correlated with OA disease stage, at an FDR of 10%. Thus, our data indicate a rise in mean serum levels of Cer class of 9.6% (5.4%; 16.2%) and of HexCer class of 4.7% (0.9%; 9.4%) per unit X OU. However, no significant association was seen in SF at the same FDR.

**Fig. 5:**
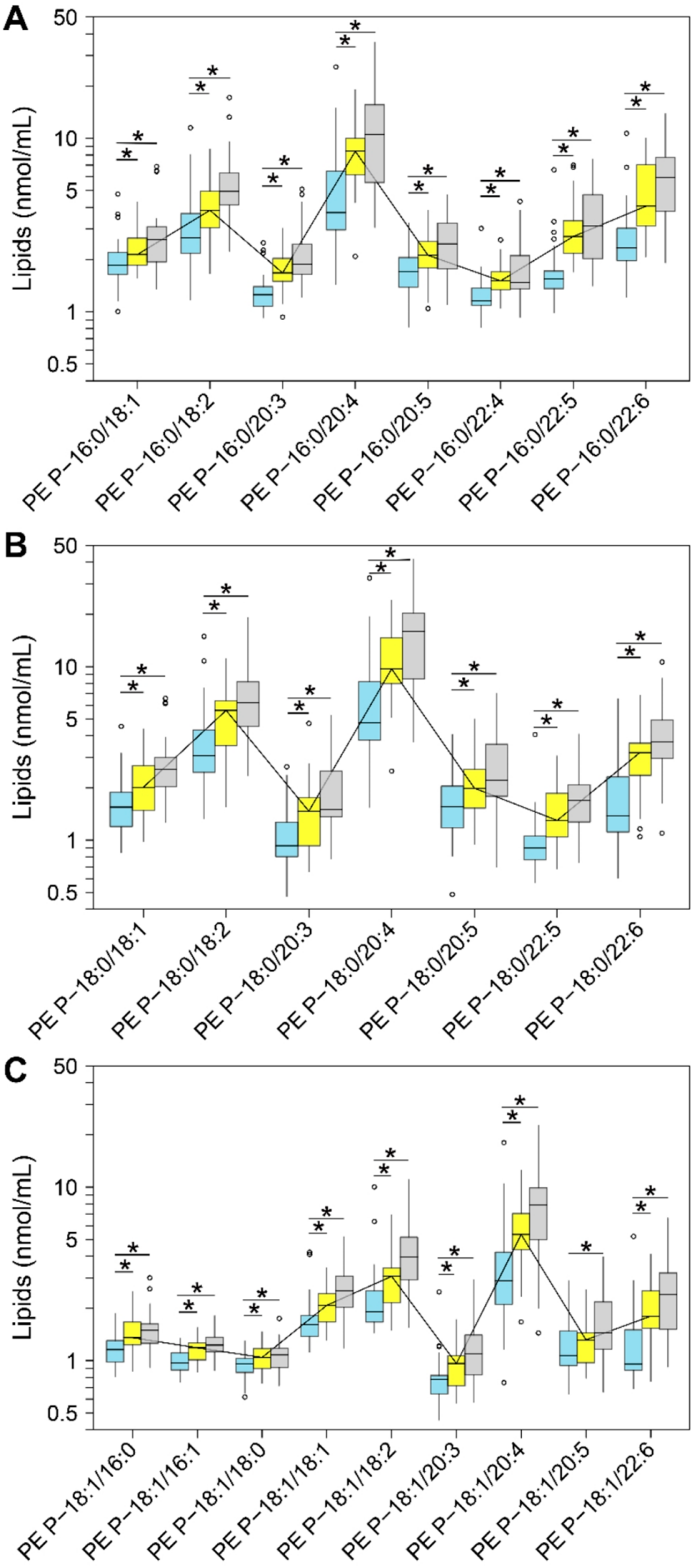
Concentrations of phosphatidylethanolamine-based plasmalogen (PE P) species in serum. ESI-MS/MS quantification in serum samples from controls (*n* = 31, blue) and early stage OA (eOA, *n* = 29, yellow) and late stage OA (lOA, *n* = 29, grey) patients. (**A)** Fatty acids (FA) with 16 carbon atoms. (**B)** FA with 18 carbon atoms. (**C)** FA with 18 carbon atoms with 1 double bond. For more information, refer to caption of Fig. 2

Next, we quantified 24 PE P species (Fig. 5, Supplementary Table S2) in serum and SF. Compared with control serum [PE P-16:0 class: 17 (15–19) nmoles/mL; PE P-18 class: 26 (24–35) nmoles/mL], the median total PE P-16:0 and PE P-18 content was higher by 1.6-fold (FDR-adj. *p* < 0.001) and 1.6-fold (FDR-adj. *p* < 0.001), respectively, in serum from patients with eOA and by 2.1-fold (FDR-adj. *p* < 0.001) and 2.3-fold (FDR-adj. *p* < 0.001) in those with lOA.

Relative to the median levels of the PE P-16.0 class [4 (3–7) nmoles/mL] and PE P-18 class [5 (4–12) nmoles/mL] in SF of the control cohort, those in patients with eOA increased by 2.2-fold (FDR-adj. *p* = 0.014) and 2.4-fold (FDR-adj. *p* = 0.037), respectively, and in patients with lOA they were 2.1-fold (FDR-adj. *p* = 0.007) and 2.4-fold (FDR-adj. *p* = 0.043) higher.

By log-linear regression analysis, there was a significant association between PE P-16 [ß = 0.08 (0.05; 0.11), *p* < 0.001], PE P-18 class [ß = 0.08 (0.05; 0.11), *p* < 0.001], and all 24 PE P species in serum and OA disease stage, even at an FDR of < 5%. These data suggest a rise in mean serum PE P-16 and PE P-18 class levels of 20.2% (12.2%; 28.8%) and 20.2% (12.2%; 28.8%), respectively, per unit X OU. We noted a significant association only for the PE P-16 class [ß = 0.05 (0.001; 0.102), *p* = 0.045] and 4 of 24 PE P species with OA progression in SF, at an FDR of < 10%. Thus, our data imply higher mean PE P-16 class levels in SF by 12.2% (0.2%; 26.5%) per unit X OU.

### Distribution of PL and SL species between serum and SF

**Fig. 6:**
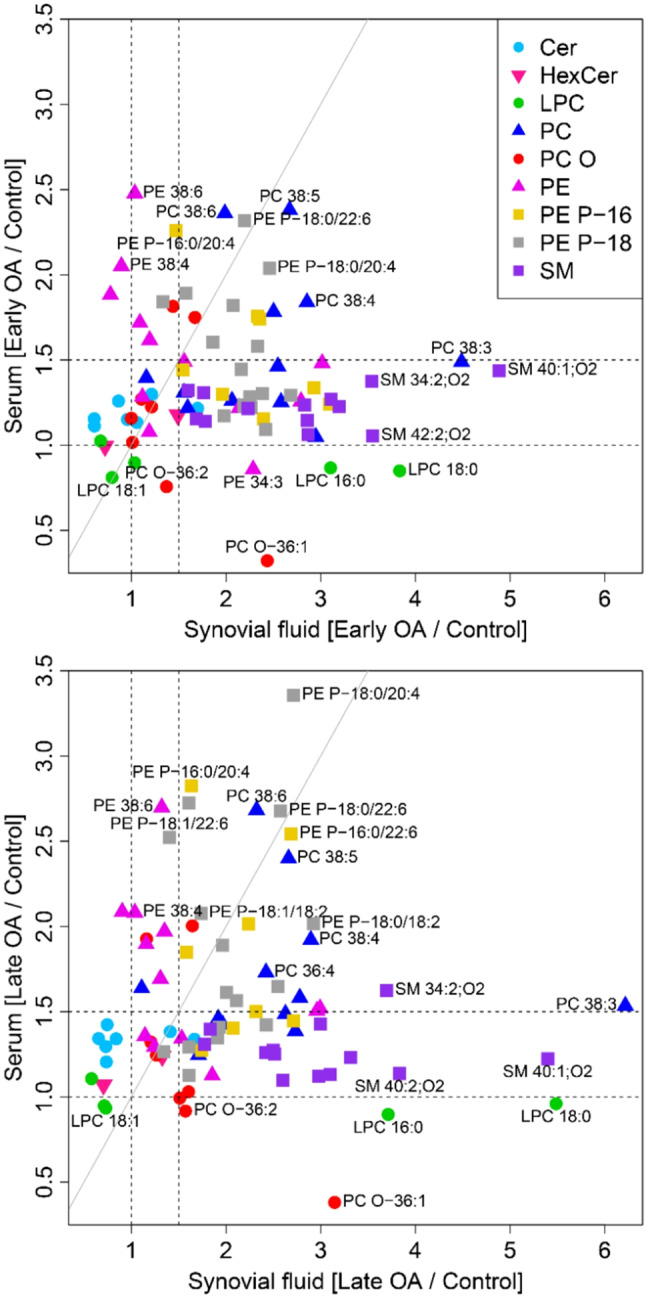
Relation between control-normalized serum and synovial fluid (SF) lipid levels per OA cohort. Ratios of lipid species concentrations between early OA and control (**A**) and between late OA and control (**B**) were calculated separately for SF and serum. The horizontal and vertical auxiliary lines at 1.5 indicate lipid species with median serum and/or SF concentrations that are at least 1.5-fold higher than their medians in healthy controls. The diagonal auxiliary line is the line of identity, above which median phospholipid or sphingolipid species concentrations are higher in serum than in SF compared with controls. Each dot represents a lipid species that is color-coded to its lipid class. Cer, ceramide; HexCer, hexosylceramide; LPC, lysophosphatidylcholine; PC, phosphatidylcholine; PC O, ether-linked phosphatidylcholine; PE, phosphatidylethanolamine; PE P, phosphatidylethanolamine-based plasmalogen; SM, sphingomyelin

To further examine the distribution of PL and SL species between the 2 compartments—serum and SF—we calculated and plotted the ratios of the median levels of all 91 lipid concentrations in eOA (Fig. 6A) and lOA (Fig. 6B), relative to their respective controls. We arbitrarily chose a threshold of 1.5 to indicate potentially clinically relevant elevations in lipid species, i.e. their median serum or SF concentrations are at least 1.5-fold higher than those of healthy controls. As shown in Fig. 6, in the eOA cohort, only 13 PL species that were higher by at least 1.5-fold in SF were also upregulated by at least 1.5-fold in serum, whereas in the lOA cohort, 23 PL species followed this pattern. Notably, 8 and 9 PL species rose by at least 1.5-fold relative to the control in the serum but not SF of eOA and lOA patients, respectively. Importantly, as the severity of OA disease increased, more lipid species were found to be elevated in serum. The concentrations of all 91 lipid species and those that were used to calculate the ratios can be found in the Supplementary Table S2.

### Serum ratios of select LPC-to-PC species

**Fig. 7:**
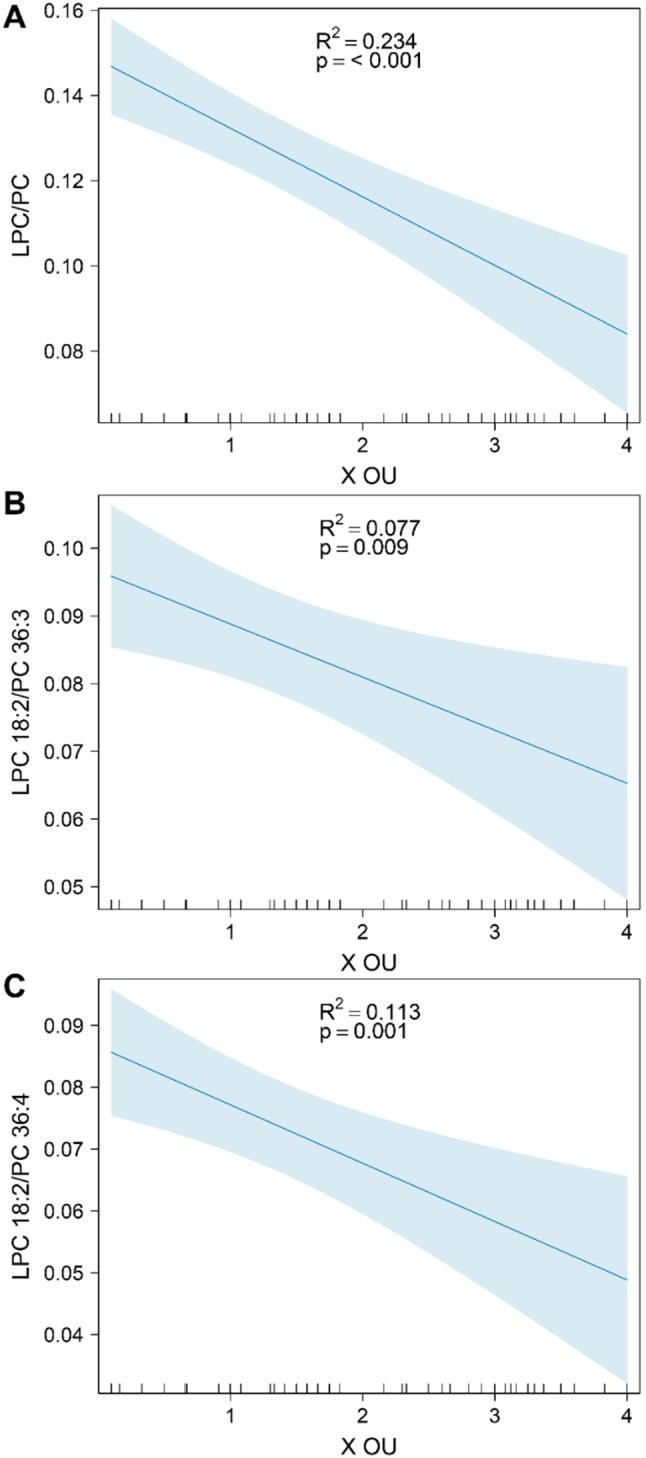
Predictor effect plots of the influence of average Outerbridge score (X OU) on the response of serum lipid ratios using linear regression models. (**A)** LPC/PC, (**B)** LPC 18:2/PC 36:3, and (**C)** LPC 18:2/PC 36:4. Multiple R^2^ and regression lines with pointwise 95% CI, indicated in blue, are shown. P-values indicate significant relations of ratios with X OU for the fitted regression model at a 5% level. CI, confidence interval; LPC, lysophosphatidylcholine; PC, phosphatidylcholine

We analyzed pooled data from cohorts of control, eOA, and lOA subjects regarding the serum ratios of LPC/PC, LPC18:2/PC36:3, and LPC18:2/PC36:4 (Fig. 7). By regression analysis, we found a significant relationship between all ratios with OA disease stage, based on X OU. However, the multiple R^2^ values suggested that only 23%, 8%, and 11% of the measured variance in the serum lipid ratios of LPC/PC, LPC 18:2/PC 36:3, and LPC 18:2/PC 36.4, respectively, were related to OA disease stage.

### General response to OA

Elevated levels of cholesterol, LDL cholesterol, triglycerides, and Apo correlated with progressive OA, increasing age, and BMI (Table 1). Thus, we were interested in whether the increases in total PLs and SLs were a component of a more general response to progressive OA, increasing age, BMI, and sex, wherein more lipids are produced and bind to elevated levels of Apo-AI and Apo B-100. By multivariate analysis of variance of the 6-dimensional general response—comprising total PLs, cholesterol, LDL cholesterol, triglycerides, and both Apo (all 6 log-transformed)—there was no significant influence of OA progression (*p* = 0.90), age (*p* = 0.90), BMI (*p* = 0.50), or sex (*p* = 0.32) separately, or of the 4 covariables jointly (Pillai´s trace test, *p* = 0.24) on the mean response vector. This finding indicates that the progression of OA, increasing age, higher BMI, and sex do not induce a significant general response in the body with regard to elevated levels of total PLs and SLs, LDL cholesterol, triglycerides, cholesterol, and both Apo.

## Discussion

This study examined the association of serum PLs and SLs with the progression of OA, as well as the relationship between these lipids in serum and SF. Our novel findings reveal marked alterations in the PL and SL profiles in human serum due to OA, with the lowest concentrations observed in healthy controls and levels consistently elevated in eOA and only slightly further elevated in lOA. Notably, even at eOA, with an X OU below 2, measurable changes in PL and SL levels were evident. Further, our new findings indicate that the serum PL and SL profiles partially mirror the elevated lipid levels that are observed in SF during OA and that the median PL and SL concentrations in serum can be 3–12 times higher than in SF.

This study validates our previous observations on the disease stage-dependent composition of PLs and SLs in human SF from postmortem controls and OA patients, expanding our analysis to a larger set of SF samples, including previously published cohorts [5, 6]. We arbitrarily considered PL and SL concentrations that were at least 1.5-fold higher than the mean levels in controls to be clinically relevant. Most PL species increased by at least 1.5-fold only in SF, not in serum, indicating a local specific response of the knee joint in OA. We observed 13 and 23 of the 91 PL species as being elevated in the SF and serum of early and late OA patients, respectively, indicating a local and systemic response. Notably, almost 10% of the PL species that were analyzed underwent an increase of at least a 1.5-fold only in the serum of OA patients compared with controls, likely reflecting a systemic response to local OA disease alone, not a systemic and local response together.

Our observations are consistent with other studies that have reported only a moderate correlation between the proteome, metabolites, and potential biomarkers in plasma with those in the SF of patients with knee OA or juvenile arthritis [26, 50, 51]. These modest associations must be taken into account if blood is to serve as a surrogate for SF in the identification of OA biomarkers. Further, our study shows that compared with the control, the increase in the concentration of lipid species is induced by OA, wherein the differential regulation of lipid species in only 1 of the 2 compartments indicates a solely systemic or local pathophysiological process.

Although cholesterol, triglyceride, LDL cholesterol, and VLDL cholesterol levels were slightly elevated in the OA cohorts and continued to increase in later OA, our statistical analysis does not support the existence of a general systemic response. Rather, it suggests a specific systemic response, potentially originating from the liver—the main organ that regulates lipid homeostasis—given that less than half of all 91 PL and SL species increased by at least 1.5-fold in the serum of OA patients. The heightened levels of these lipid species may be driven by increased synthesis, modulation of catabolism, and clearance.

Our data demonstrate that depending on the lipid class, PL and SL levels in serum are generally 3–12 times higher than in SF. Although acute exercise can elicit stress-induced changes in the levels of FAs and certain PL species in SF, suggesting their mechanosensitivity, the PLs and SLs that are elevated only in serum likely do not originate from FLSs [52]. PLs and SLs can be actively synthesized and secreted into the SF by articular cells, such as FLSs [13, 14, 17]. However, it is unlikely that PLs and SLs that are derived from FLSs are primarily responsible for the large amount of PL and SL species required to generate disease-specific signatures in blood with its large volume.

Instead, SF lipids may derive primarily from blood vessels in the synovial capsule, with their diffusion influenced by such factors as blood concentration, synovial vascular density, vessel size, inflammation-induced vascular permeability, and low lipoprotein concentrations in SF compared with blood [22, 53, 54, 55]. For instance, Oliviero et al. [53] reported a 3.3-fold higher level of Apo-AI in OA serum (170 mg/dL) than in paired SF (52 mg/dL), whereas our analysis revealed a 7.7-fold greater median level of Apo B-100 in serum (90 mg/dL) versus SF (12 mg/dL) in healthy controls [40]. Both Apo gradients could have contributed substantially to the concentration gradients of PL and SL species that were observed in our study between blood and SF. By Pearson correlation, serum levels of Apo-AI (*r* = 0.24; *p* = 0.041) and especially Apo B-100 (*r* = 0.44; *p* < 0.001) were moderately related to OA disease stage, providing the lipoproteins, which are necessary for transporting elevated PL, SL, and cholesterol levels. Notably, Apo-AI levels in SF, but not serum, are negatively associated with increasing cartilage destruction, according to a recent study [56]. These incongruent findings highlight the complexity of the systemic response to OA, warranting further investigation into its underlying mechanisms.

Detecting changes in PL levels in the SF of eOA patients is crucial for understanding the disease and developing treatments. The American College of Rheumatology (ACR) diagnostic criteria, however, are unlikely to recruit eOA patients, who are often aged under 50 years and may have developed osteophytes and visible cartilage abnormalities [5, 57, 58]. Thus, as reported in previous studies [5, 6], we classified eOA based on the macroscopic X OU. As the disease progressed, we observed an increase in the number of PL and SL species that were elevated in serum—a trend that was noticeable even in lOA compared with eOA stages. Despite similar systemic CRP levels in the eOA and lOA cohorts, the stage-dependent association with specific PLs suggests a local increase in inflammation-induced synovial vascular permeability, facilitating the diffusion of lipoproteins from blood vessels to SF [55]. Given that the liver supplies a large proportion of plasma lipids, the hepatocytes may have contributed significantly to the specific changes in lipid profiles, which were observed in blood and, to some extent, in joint SF.

In blood, LPCs are formed from PC by lecithin-cholesterol acyltransferase (LCAT), whereas in tissues, this process is mediated primarily by phospholipase A_2_. Previous studies have reported higher LPC/PC, LPC 18:2/PC 44:3, and LPC 16:0/PC 38:2 ratios and lower levels of PC 38:2 and PC 40:3 in the plasma and serum of patients with knee OA and endotypes of OA, respectively, compared with controls, using a commercial kit [59–61]. In our study, we also calculated the LPC/PC ratio in the serum of OA patients, as well as LPC 18:2/PC 36:3 and LPC 18:2/PC 36:4 ratios. We chose the latter 2 ratios due to the chemical similarity of these species with those of the LPC 18:2/PC 44:3 ratio, as reported by Zhai et al. [59]; we excluded PC 44:3, PC 38:2, and PC 40:3 from further analysis due to their low levels (less than 1% of the median of the total corresponding PC class). Our serum LPC/PC ratios and the 2 selected ratios correlated negatively with the severity of OA, based on the nearly constant total LPC and LPC 18:2 serum levels in our 3 cohorts, allowing us to exclude the possibility of any elevations in LCAT and phospholipase A_2_; similarly, Zhai et al. [59] determined no increase in LCAT in the serum of OA patients by ELISA.

However, our significantly but slightly decreased ratios are in contrast to reports that have described an association between higher plasma LPC/PC and serum LPC 18:2/PC44:3 ratios with knee OA and volume of loss of cartilage, respectively, using a commercial kit [59, 61]. Using the same kit, no OA-induced alterations in LPC/PC ratio could be detected by Tootsi et al. [62], and a slight, albeit insignificant, increase in plasma LPC (16:0 + 18:0)/PC (36:4 + 38:4) ratio was observed in patients of the Dutch CHeCK cohort, which had early and moderate OA [63]. The elevated plasma levels of PC 34:3 and PC 36:3 in Zhang et al. [64] confirm our findings regarding elevated serum levels for a variety of PC species. Rockel et al. [65] also found that individual LPC and PC species can indicate OA in plasma with a certain degree of accuracy. In conclusion, the calculation of ratios of several PLs that can be reproducibly quantified is a promising approach to identify biomarkers for the various OA endotypes.

Limitations of our study include its analysis of nonfasting serum, unknown lipid diet, and small sample size, given the complexity of OA patients and the disease per se. SF was obtained from eOA and lOA patients after anesthesia, which may have altered the permeability of blood and lymphatic vessels, in turn influencing PL and SL levels in SF. Furthermore, it cannot be ruled out that, in addition to the OA knee joints, the patients also had other joints affected by OA that were not diagnosed due to their asymptomatic nature, which contributed to the elevated PL and SL serum levels. There was a discrepancy in age between the healthy control group and the OA participants, who also displayed an increase in BMI. However, PL and SL levels in serum showed no significant Pearson correlation with age or BMI, and including them as confounders in regression models would introduce problematic multicolinearity without established evidence of their influence on PL or SL synthesis in healthy joints. In our study, we determined 91 lipid species of 6 classes in the SF and serum, although the exact number depends on the analytical techniques used and the resolution of the lipidomics methods applied. The same lipid extraction protocol was used for both SF and serum, resulting in similar lipidomes. The Bligh and Dyer [41] lipid extraction used is a very efficient lipid extraction covering a wide range of lipid classes and does not change the lipid class ratios, especially when the recovery is monitored by adding internal standards prior to extraction as in our study. This study, comprising partially paired serum and SF samples from eOA and lOA patients and healthy controls, grouped for ethical reasons, should be regarded as exploratory, warranting validation in diverse and larger cohorts. In addition, future studies need to determine the cause and effect of higher PL and SL concentrations in serum and SF.

## Conclusions

Our lipidomic study is a comprehensive view of the distribution of PLs and SLs in serum and SF, revealing a strong association between specific PLs and SLs and the progression of OA. Human serum PLs and SLs partly mirror the alterations in knee SF of the same OA patients compared with healthy controls. With nearly 10% of quantified PL species elevated exclusively in serum during OA, our findings suggest an additional systemic response to the local OA disease. As the significant changes in serum levels of PLs and SLs were already measured in early stages of the disease with an OU score below 2, our data implicate potential novel lipid biomarkers even for the very early, non-radiologically detectable stages of OA. Impaired lipid metabolism, with elevated levels of PLs and SLs, is a pathophysiological hallmark of OA, necessitating further study to determine the functions of each species and the significance of their altered levels.

## Electronic supplementary material

Below is the link to the electronic supplementary material.

Supplementary Material 1

## Data Availability

All chemicals used as well as clinical and lipidomic data of all samples are provided in Methods, Table 1, Supplementary Tables S1 and S2 (Additional file 1). De-identified data used in this study may be obtained upon a reasonable request to the corresponding author, as long as the request is evaluated as scientifically relevant and pertinent.

## Abbreviations

ANOVA: Analysis of variance
Apo: Apolipoprotein
ß: Regression coefficient
BMI: Body mass index
Cer: Ceramide
CRP: C-reactive protein
eOA: Early stage osteoarthritis
ESI-MS/MS: Electrospray ionization tandem mass spectrometry
FA: Fatty acid
FLS: Fibroblast-like synoviocytes
FDR: False discovery rate
HexCer: Hexosylceramide
HDL: High-density lipoprotein
LDL: Low-density lipoprotein
lOA: Late stage osteoarthritis
LCAT: Lecithin-cholesterol acyltransferase
LPC: Lysophosphatidylcholine
OA: Osteoarthritis
OU: Outerbridge score
PE: Phosphatidylethanolamine
PE P: Phosphatidylethanolamine-based plasmalogen
PL: Phospholipid
PC: Phosphatidylcholine
PC O: Ether-linked phosphatidylcholine
RA: Rheumatoid arthritis
SF: Synovial fluid
SL: Sphingolipid
VLDL: Very-low-density lipoprotein
X OU: Average Outerbridge score
