# Supplementary material for: Serum phospholipids and sphingolipids are linked to early-stage osteoarthritis by lipidomic profiling
Source: Arthritis Res Ther. 2025 Mar 31;27:69. doi: 10.1186/s13075-025-03537-4 (PMC11956431; doi:10.1186/s13075-025-03537-4)
Supplement: Supplementary file 1 — Supplementary Material 1 [file 13075_2025_3537_MOESM1_ESM.pdf]

SUPPLEMENTARY MATERIAL

Serum phospholipids and sphingolipids are linked to early-stage osteoarthritis by lipidomic profiling

Gerrit Eichner[1], Gerhard Liebisch[2], Christiane Hild[3],
Markus Rickert[3] and Juergen Steinmeyer[3*]

[1]Mathematical Institute, Justus Liebig University Giessen, Giessen, Germany. [2]Department of Clinical Chemistry and Laboratory Medicine, University Hospital Regensburg, Regensburg, Germany. [3]Laboratory for Experimental Orthopaedics, Department of Orthopaedics and Orthopaedic Surgery, Justus Liebig University Giessen, Giessen, Germany.

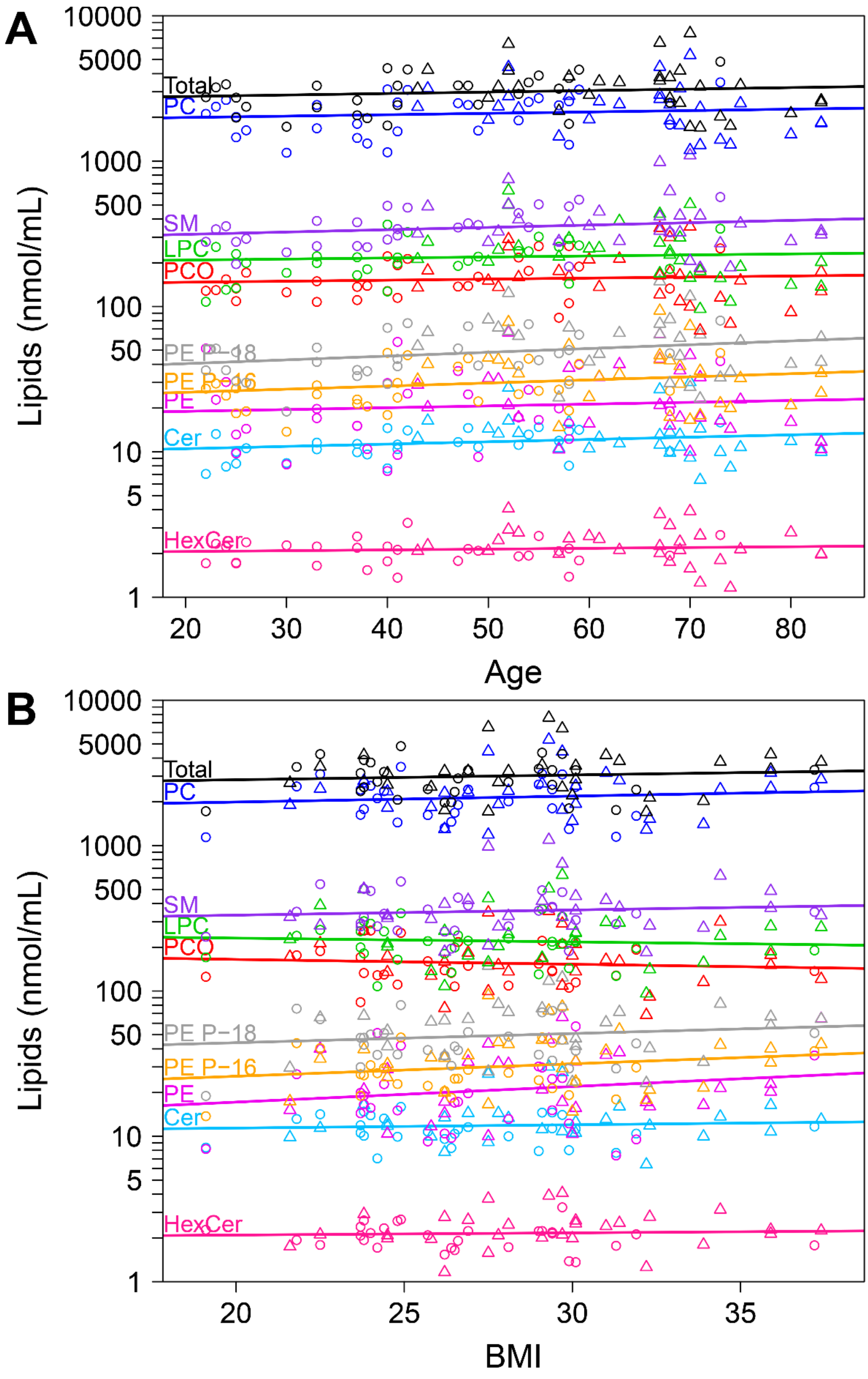

Supplementary Fig. 1 Relationship between the levels of lipid classes and (**A**) age and (**B**) BMI. Data for all early (open circle) and late (open triangle) stage OA patients in serum (n = 58) are presented with class-wise regression lines. Pearson´s correlation between level of lipid class and age or BMI always ranged between -0.07 and +0.24, and all of their corresponding FDR-adjusted p-values were above 0.3.

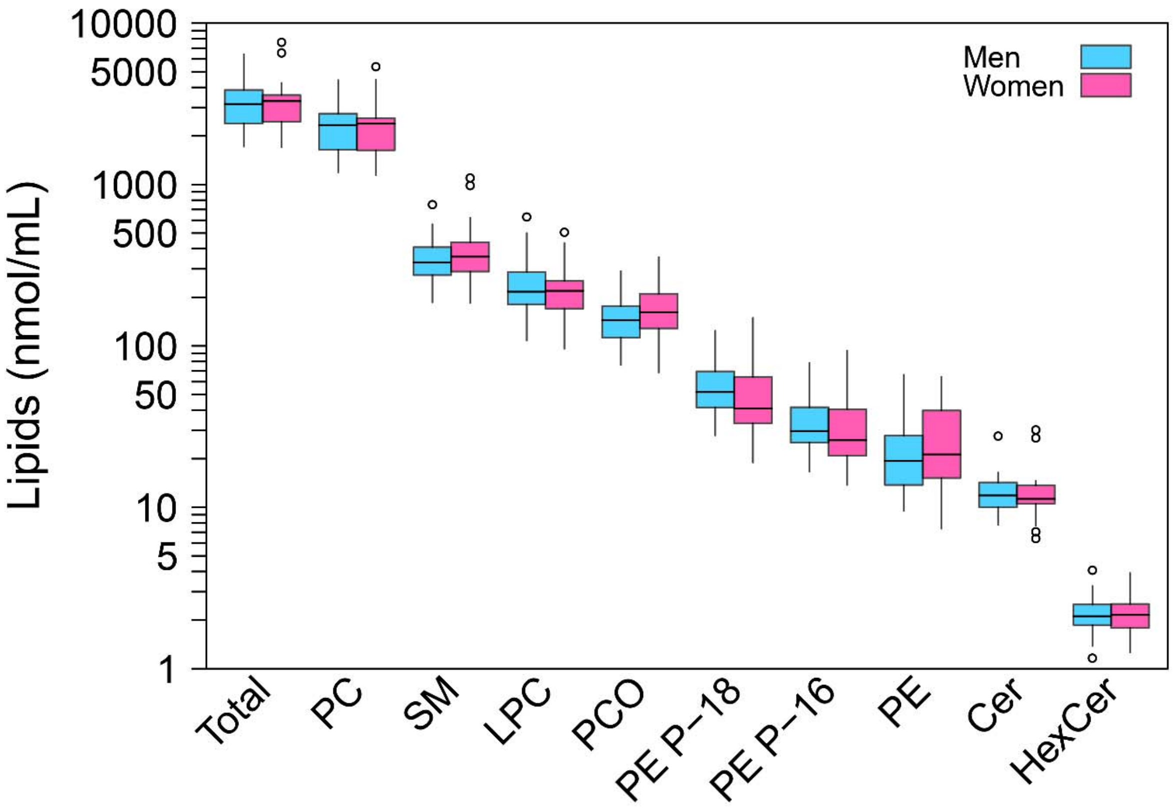

Supplementary Fig. 2 Relationship between the levels of lipid classes and sex. Data for all early and late stage OA patients in serum (*n* = 58) are presented as boxplots with median, interquartile range and outliers for male (*n* = 32) and female (*n* = 26) sex, with FDR-adjusted p-values of Wilcoxon´s rank sum test always above 0.7.

Supplementary Table 1. Concentrations of human phospholipid and sphingolipid classes in serum and synovial fluid.

Class	Serum (Se) Synovial fluid (SF)	Median Control [nmol/mL]	Q1 Control [nmol/mL]	Q3 Control [nmol/mL]	Median eOA [nmol/mL]	Q1 eOA [nmol/mL]	Q3 eOA [nmol/mL]	FDR-adj. p-value C-eOA	Median lOA [nmol/mL]	Q1 lOA [nmol/mL]	Q3 lOA [nmol/mL]	FDR-adj. p-value C-lOA	FDR-adj. p-value eOA-lOA
Cer	Se	8.99	8.22	10.35	11.15	9.93	13.26	0.013	12.45	10.59	14.45	<0.001	0.283
Cer	SF	1.8	1.37	2.59	1.6	1.25	2.39	0.893	1.88	1.57	2.35	0.83	0.877
Cer	Se/SF	4.99	6	3.99	6.97	7.94	5.54	0.693	6.64	6.73	6.14	0.48	0.971
HexCer	Se	1.89	1.67	2.19	2.09	1.76	2.23	0.245	2.25	1.99	2.66	0.009	0.283
HexCer	SF	0.42	0.35	0.54	0.4	0.24	0.52	0.524	0.4	0.27	0.46	0.382	0.877
HexCer	Se/SF	4.53	4.83	4.06	5.27	7.23	4.28	0.437	5.56	7.37	5.82	1	0.971
LPC	Se	240.1	190.9	297.9	217.5	180.5	256.9	0.111	226.99	175.41	279.06	0.504	0.483
LPC	SF	23.35	13.88	42.63	53.85	23.02	95.83	0.014	65.86	40.58	98.42	<0.001	0.877
LPC	Se/SF	10.28	13.76	6.99	4.04	7.84	2.68	0.002	3.45	4.32	2.84	0.001	0.971
PC	Se	1503.3	1228.4	2010.9	2115.6	1622.4	2553.8	0.006	2361.8	1825.3	2792.1	<0.001	0.483
PC	SF	170.9	97.58	196.5	375.7	138.2	546.58	0.014	380.57	280.23	532.92	<0.001	0.877
PC	Se/SF	8.79	12.59	10.23	5.63	11.74	4.67	0.009	6.21	6.51	5.24	0.002	0.971
PCO	Se	161.8	131.2	213.3	138.4	127.8	188.03	0.245	160.33	127.08	183.53	0.659	0.527
PCO	SF	13.59	9.69	20.32	29.34	13.47	54.62	0.017	35.73	26.66	57.55	<0.001	0.877
PCO	Se/SF	11.91	13.53	10.49	4.72	9.49	3.44	<0.001	4.49	4.77	3.19	<0.001	0.971
PE	Se	11.7	8.42	17.02	17.27	13.15	26.73	0.015	21.01	16.09	32.69	<0.001	0.289
PE	SF	1.77	1.12	4.72	2.55	2.08	3.58	0.808	2.8	1.76	3.66	0.763	0.877
PE	Se/SF	6.6	7.5	3.61	6.76	6.32	7.47	0.873	7.49	9.14	8.92	1	0.971

PE P-16	Se	16.71	14.63	19.45	26.29	22.23	30.84	<0.001	34.64	23.75	43.07	<0.001	0.283
PE P-16	SF	3.74	2.71	6.66	8.4	6.59	10.86	0.014	7.81	6.61	10.45	0.007	0.877
PE P-16	Se/SF	4.46	5.39	2.92	3.13	3.37	2.84	0.139	4.43	3.59	4.12	0.292	0.971
PE P-18	Se	26.23	23.51	34.77	42.7	36.33	51.41	<0.001	61.33	41.65	71.59	<0.001	0.283
PE P-18	SF	5.36	4.2	11.78	13.18	10.15	15.78	0.037	12.67	10.1	16.21	0.043	0.877
PE P-18	Se/SF	4.89	5.59	2.95	3.24	3.58	3.26	0.189	4.84	4.12	4.42	0.398	0.971
SM	Se	277.52	235.44	341.43	341.08	278.4	388	0.038	332.11	282.31	422.44	0.009	0.527
SM	SF	39.08	26.71	53.52	94.58	45.33	160.01	0.017	100.42	72.17	144.53	0.001	0.877
SM	Se/SF	7.1	8.81	6.38	3.61	6.14	2.42	0.009	3.31	3.91	2.92	0.005	0.971
Total PLs	Se	2292.5	1880.7	2964.0	2898.6	2368.6	3377.7	0.022	3254.1	2536.8	3771.8	0.002	0.483
Total PLs	SF	278.45	191.34	364.75	564.75	249.14	867.57	0.014	606.38	446.77	865.98	<0.001	0.877
Total PLs	Se/SF	8.23	9.83	8.13	5.13	9.51	3.89	0.006	5.37	5.68	4.36	0.001	0.971

Supplementary Table 1: FDR-adj. p-values of Wilcoxon´s rank sum test for serum and SF data, and of custom-made test for Se/SF ratio data; adj., adjusted; C, control; Cer, ceramide; eOA, early stage osteoarthritis; HexCer, hexosylceramide; lOA, late stage osteoarthritis; LPC, lysophosphatidylcholine; PC, phosphatidylcholine; PC O, ether-linked phosphatidylcholine; PE, phosphatidylethanolamine; PE P, phosphatidylethanolamine-based plasmalogen; PL, phospholipid; Se, serum; SF, synovial fluid; Q, quartile; SM, sphingomyelin.

Supplementary Table 2. Concentrations of human phospholipid and sphingolipid species in serum and synovial fluid.

Specie	Serum (Se) Synovial fluid (SF)	Median Control [nmol/mL]	Q1 Control [nmol/mL]	Q3 Control [nmol/mL]	Median eOA [nmol/mL]	Q1 eOA [nmol/mL]	Q3 eOA [nmol/mL]	FDR-adj. p-value C-eOA	Median lOA [nmol/mL]	Q1 lOA [nmol/mL]	Q3 lOA [nmol/mL]	FDR-adj. p-value C-lOA	FDR-adj. p-value eOA - lOA
Cer 18:1;O2/16:0	Se	0.76	0.64	0.9	0.88	0.81	0.96	0.044	1.02	0.81	1.2	0.003	0.209
Cer 18:1:O2/16:0	SF	0.33	0.21	0.52	0.2	0.17	0.34	0.147	0.22	0.19	0.27	0.079	0.944
Cer 18:1;O2/16:0	Se/SF	2.28	3.01	1.74	4.34	4.9	2.82	0.015	4.68	4.37	4.39	0.001	0.151
Cer 18:1;O2/18:0	Se	0.57	0.53	0.63	0.66	0.6	0.7	0.008	0.74	0.61	0.83	<0.001	0.103
Cer 18:1;O2/18:0	SF	0.11	0.06	0.17	0.11	0.07	0.14	0.818	0.08	0.05	0.11	0.194	0.478
Cer 18:1;O2/18:0	Se/SF	5.08	9.32	3.74	6.1	9.03	5.1	0.035	9	11.88	7.66	<0.001	0.066
Cer 18:1;O2/20:0	Se	0.58	0.52	0.66	0.66	0.6	0.77	0.013	0.7	0.61	0.84	0.004	0.387
Cer 18:1;O2/20:0	SF	0.09	0.06	0.11	0.1	0.06	0.13	0.462	0.07	0.04	0.1	0.489	0.478
Cer 18:1;O2/20:0	Se/SF	6.46	9.41	5.74	6.91	10.26	5.99	0.144	10.58	13.94	8.74	0.003	0.151
Cer 18:1;O2/22:0	Se	1.36	1.15	1.6	1.66	1.47	1.91	0.013	1.82	1.53	2.27	<0.001	0.209
Cer 18:1;O2/22:0	SF	0.15	0.12	0.21	0.25	0.17	0.33	0.109	0.24	0.21	0.3	0.012	0.96
Cer 18:1;O2/22:0	Se/SF	9.38	9.45	7.81	6.71	8.56	5.71	0.007	7.53	7.37	7.51	<0.001	0.213
Cer 18:1;O2/23:0	Se	1.2	1.04	1.4	1.51	1.14	1.73	0.044	1.6	1.46	1.89	<0.001	0.103
Cer 18:1;O2/23:0	SF	0.24	0.16	0.31	0.21	0.13	0.33	0.83	0.2	0.18	0.29	0.667	0.988
Cer 18:1;O2/23:0	Se/SF	4.94	6.46	4.52	7.19	8.92	5.19	0.091	7.89	8.17	6.62	0.001	0.151
Cer 18:1;O2/24:0	Se	2.87	2.39	3.4	3.72	2.89	4.4	0.031	3.96	3.46	4.68	<0.001	0.217
Cer 18:1;O2/24:0	SF	0.39	0.31	0.56	0.48	0.31	0.88	0.439	0.55	0.47	0.7	0.147	0.944
Cer 18:1;O2/24:0	Se/SF	7.31	7.79	6.1	7.81	9.19	4.98	0.175	7.16	7.36	6.64	0.014	0.49

Cer 18:1;O2/24:1	Se	1.7	1.43	2.04	1.89	1.69	2.51	0.044	2.42	2.01	2.91	<0.001	0.103
Cer 18:1;O2/24:1	SF	0.51	0.37	0.71	0.31	0.23	0.49	0.147	0.38	0.32	0.55	0.173	0.944
Cer 18:1;O2/24:1	Se/SF	3.35	3.86	2.87	6.11	7.28	5.09	0.477	6.41	6.17	5.32	0.55	0.18
HexCer18:1;O2/16:0	Se	0.84	0.71	1.01	0.99	0.83	1.12	0.044	1.04	0.92	1.27	0.004	0.209
HexCer 18:1;O2/16:0	SF	0.12	0.11	0.16	0.18	0.11	0.3	0.147	0.16	0.13	0.23	0.147	0.944
HexCer 18:1;O2/16:0	Se/SF	6.88	6.68	6.31	5.43	7.38	3.75	0.091	6.42	7.28	5.6	0.004	0.18
HexCer 18:1;O2/24:1	Se	1.03	0.95	1.17	1.03	0.9	1.2	0.725	1.11	1.06	1.42	0.066	0.103
HexCer 18:1;O2/24:1	SF	0.3	0.27	0.39	0.22	0.12	0.28	0.044	0.21	0.16	0.29	0.012	0.944
HexCer 18:1;O2/24:1	Se/SF	3.42	3.5	3.02	4.71	7.8	4.25	0.477	5.18	6.81	4.89	0.266	0.151
LPC 16:0	Se	151.87	116.42	184.34	131.7	108.69	162.32	0.223	136.26	105.39	179.05	0.944	0.658
LPC 16:0	SF	11.02	6.36	19.03	34.23	13.8	58.76	0.001	40.93	25.96	58.85	<0.001	0.71
LPC 16:0	Se/SF	13.78	18.31	9.69	3.85	7.87	2.76	0.001	3.33	4.06	3.04	0.001	0.819
LPC 18:0	Se	62.29	47.36	80.17	52.94	44.13	60.24	0.196	59.82	43.36	68.43	0.944	0.464
LPC 18:0	SF	3.43	2	4.57	13.16	5.72	24.97	<0.001	18.82	10.55	24.04	<0.001	0.71
LPC 18:0	Se/SF	18.16	23.64	17.53	4.02	7.71	2.41	0.001	3.18	4.11	2.85	0.001	0.819
LPC 18:1	Se	12.42	9.99	16.03	10.07	8.51	14.47	0.231	11.6	10.15	16.08	0.988	0.464
LPC 18:1	SF	4.42	2.17	6.66	3.53	1.5	6.19	0.591	3.23	2.03	4.66	0.28	0.829
LPC 18:1	Se/SF	2.81	4.6	2.41	2.86	5.66	2.34	0.868	3.59	5.01	3.45	0.453	0.819
LPC 18:2	Se	12.22	9.84	16.35	10.97	8.12	15.42	0.231	11.58	9	17.72	0.988	0.464
LPC 18:2	SF	3.17	1.84	7.2	3.29	1.43	5.66	0.591	2.27	1.54	3.67	0.199	0.71
LPC 18:2	Se/SF	3.85	5.36	2.27	3.34	5.69	2.72	0.868	5.11	5.85	4.83	0.284	0.819
LPC 20:4	Se	3.31	2.69	4.25	3.4	2.92	4.21	0.607	3.67	2.48	5.17	0.988	0.914
LPC 20:4	SF	1.4	1.08	2.14	0.95	0.52	1.87	0.162	0.81	0.52	1.37	0.047	0.71
LPC 20:4	Se/SF	2.37	2.49	1.99	3.59	5.64	2.25	0.571	4.52	4.79	3.76	0.048	0.819

PC 32:0	SE	17.17	14.46	21.89	20.92	15.6	26.33	0.053	21.38	17.41	25.7	0.023	0.793
PC 32:0	SF	5.97	5.13	9.31	9.53	4.11	19.83	0.244	10.22	8.64	15.16	0.061	0.963
PC 32:0	Se/SF	2.87	2.82	2.35	2.19	3.79	1.33	0.458	2.09	2.02	1.7	0.264	0.986
PC 32:1	Se	16.86	13.74	21.63	23.53	18.23	36.59	0.022	27.62	15.9	37.19	0.017	0.793
PC 32:1	SF	3.6	2.27	4.98	4.18	1.82	6.77	0.872	4	2.36	6.68	0.809	0.963
PC 32:1	Se/SF	4.68	6.05	4.34	5.64	10.02	5.41	0.47	6.91	6.73	5.57	0.419	0.986
PC 34:1	Se	253.08	215.82	332.12	319.18	244.53	424.66	0.052	369.35	246.56	426.29	0.023	0.793
PC 34:1	SF	31.52	20.95	46.58	65.06	26.33	93.53	0.055	60.49	43.48	81.32	0.004	0.963
PC 34:1	Se/SF	8.03	10.3	7.13	4.91	9.29	4.54	0.066	6.11	5.67	5.24	0.027	0.986
PC 34:2	Se	429.46	334.74	602.73	561.88	465.51	652.48	0.048	611.53	473.56	686.72	0.009	0.793
PC 34:2	SF	37.91	16.63	40.12	59.03	31.89	102.71	0.015	74.53	53.56	92.4	<0.001	0.963
PC 34:2	Se/SF	11.33	20.13	15.03	9.52	14.6	6.35	0.017	8.2	8.84	7.43	0.003	0.986
PC 36:1	Se	54.07	45.61	66.53	56.7	47.67	80.19	0.257	74.98	51.58	96.38	0.01	0.793
PC 36:1	SF	5.83	4.03	9.1	17.21	6.39	21.33	0.024	15.94	10.99	20.56	<0.001	0.963
PC 36:1	Se/SF	9.27	11.32	7.31	3.29	7.47	3.76	0.01	4.7	4.69	4.69	0.002	0.986
PC 36:2	Se	239.89	197.95	311.72	300.77	247.32	402.74	0.033	357	308.81	423.98	0.001	0.793
PC 36:2	SF	22.91	12.87	32.16	59.15	24.59	93.43	0.003	60.21	47.2	84.15	<0.001	0.963
PC 36:2	Se/SF	10.47	15.38	9.69	5.09	10.06	4.31	0.003	5.93	6.54	5.04	<0.001	0.986
PC 36:3	Se	121.16	93.55	154.77	177.32	131.58	210.78	0.013	191.68	134.01	215.16	0.002	0.793
PC 36:3	SF	10.64	5.46	13.04	27.1	11.42	45.9	0.003	29.57	21.07	38.85	<0.001	0.963
PC 36:3	Se/SF	11.39	17.15	11.87	6.54	11.52	4.59	0.003	6.48	6.36	5.54	0.001	0.986
PC 36:4	Se	130.14	106.96	185.68	232.13	149.78	282.81	0.001	225.22	171.43	309.44	0.001	0.793
PC 36:4	SF	13.59	8.82	21.09	34.01	13.61	66.73	0.005	32.92	21.64	50.92	<0.001	0.963
PC 36:4	Se/SF	9.58	12.13	8.8	6.83	11	4.24	0.02	6.84	7.92	6.08	0.009	0.986

PC 38:3	Se	44.64	34.3	52.72	66.47	45.97	84.83	0.003	68.55	51.39	80.29	0.001	0.793
PC 38:3	SF	2.67	2.03	4.99	11.97	8.48	20.26	<0.001	16.58	11.27	22.72	<0.001	0.963
PC 38:3	Se/SF	16.74	16.9	10.56	5.55	5.42	4.19	0.02	4.13	4.56	3.53	0.016	0.986
PC 38:4	Se	80.71	66.63	112.3	148.41	109.79	183.29	<0.001	155.07	114.81	221.37	<0.001	0.793
PC 38:4	SF	12.79	9.62	17.97	36.53	17.52	59.54	0.002	37.05	25.44	49.97	<0.001	0.963
PC 38:4	Se/SF	6.31	6.93	6.25	4.06	6.27	3.08	0.01	4.19	4.51	4.43	0.006	0.986
PC 38:5	Se	31.5	27.59	52.81	74.96	44.81	87.97	<0.001	75.56	46.92	90.39	<0.001	0.793
PC 38:5	SF	4.85	3.68	8.33	12.96	6.53	26.04	0.002	12.91	8.82	19.38	<0.001	0.963
PC 38:5	Se/SF	6.49	7.49	6.34	5.78	6.86	3.38	0.02	5.85	5.32	4.66	0.016	0.986
PC 38:6	Se	33.88	26.02	63.41	80	45.46	112.72	<0.001	90.91	61.47	116.25	<0.001	0.793
PC 38:6	SF	5.01	2.34	5.87	9.97	5.13	20.87	0.004	11.65	7.65	18.98	<0.001	0.963
PC 38:6	Se/SF	6.76	11.12	10.8	8.03	8.86	5.4	0.027	7.81	8.03	6.12	0.016	0.986
PC O-32:0	Se	4.34	2.42	4.82	4.41	3.18	5.47	0.162	4.31	3.05	5.97	0.228	1
PC O-32:0	SF	0.81	0.6	1.2	0.82	0.41	2.41	0.845	1.22	0.59	1.79	0.395	0.841
PC O-32:0	Se/SF	5.38	4.03	4.02	5.41	7.76	2.27	0.646	3.53	5.16	3.34	0.747	0.916
PC O-32:1	Se	2.74	2.22	3.64	3.49	2.33	4.71	0.162	3.41	2.38	5.29	0.084	0.918
PC O-32:1	SF	0.85	0.64	1.08	0.94	0.38	1.71	0.845	1.08	0.48	1.67	0.605	0.894
PC O-32:1	Se/SF	3.23	3.49	3.37	3.7	6.18	2.76	0.646	3.17	4.91	3.16	0.747	0.916
PC O-34:1	Se	10.11	8.4	12.83	12.39	10.27	15.57	0.075	10.42	8.21	16.56	0.484	0.918
PC O-34:1	SF	2.08	1.95	3.26	2.53	1.88	5.37	0.505	3.35	2.48	4.73	0.12	0.841
PC O-34:1	Se/SF	4.85	4.3	3.93	4.89	5.47	2.9	0.941	3.11	3.3	3.5	0.572	0.916
PC O-34:2	Se	10.78	8.24	12.79	12.49	10.51	16	0.065	14.24	10.89	18.29	0.025	0.918
PC O-34:2	SF	1.97	1.46	3.65	1.97	1.4	3.15	0.845	2.38	2.03	3.75	0.395	0.841
PC O-34:2	Se/SF	5.47	5.64	3.5	6.33	7.51	5.07	0.646	5.98	5.36	4.88	0.862	0.916

PC O-36:1	Se	34.79	20.28	49.85	11.22	8.94	22.56	<0.001	13.26	9.66	30.2	0.001	0.932
PC O-36:1	SF	0.97	0.5	1.33	2.35	0.65	5.67	0.239	3.04	2.33	5.39	<0.001	0.841
PC O-36:1	Se/SF	36	40.18	37.36	4.77	13.85	3.98	0.646	4.36	4.15	5.61	0.572	0.916
PC O-36:2	Se	16.02	11.9	20.24	12.13	11.13	18.65	0.21	14.7	10.77	21.34	0.547	0.918
PC O-36:2	SF	1.45	0.93	2.03	2	0.98	3.58	0.469	2.29	1.65	3.35	0.028	0.841
PC O-36:2	Se/SF	11.02	12.82	9.97	6.08	11.37	5.21	0.646	6.43	6.52	6.36	0.572	0.916
PC O-36:3	Se	6.34	4.9	7.34	7.23	6.6	9.4	0.051	8	5.6	10.49	0.025	0.918
PC O-36:3	SF	NA	NA	NA	0.96	0.34	2.26	NA	1.21	0.74	2.11	NA	0.841
PC O-36:3	Se/SF	NA	NA	NA	7.55	19.15	4.17	NA	6.62	7.56	4.96	NA	0.916
PC O-36:4	Se	8.9	8.05	13.4	15.57	11.77	20.34	<0.001	17.83	11.03	23.84	0.001	0.918
PC O-36:4	SF	1.84	1.66	2.55	3.08	1.65	5.31	0.437	3.03	1.91	5.24	0.12	0.894
PC O-36:4	Se/SF	4.84	4.85	5.26	5.06	7.14	3.83	0.829	5.89	5.76	4.55	0.572	0.916
PC O-36:5	Se	6.05	5.33	8.14	10.97	8.1	13.38	<0.001	11.65	7.89	15.86	0.001	0.918
PC O-36:5	SF	2.37	1.68	3.23	3.41	1.68	5.26	0.505	2.75	1.82	4.47	0.395	0.894
PC O-36:5	Se/SF	2.55	3.17	2.52	3.22	4.82	2.54	0.646	4.24	4.35	3.55	0.747	0.916
PC O-38:2	Se	14.29	7.3	20.98	2.41	1.42	7.89	0.001	2.46	1.55	7.51	0.001	1
PC O-38:2	SF	NA	NA	NA	1.02	0.11	2.7	NA	1.09	0.52	1.98	NA	0.841
PC O-38:2	Se/SF	NA	NA	NA	2.37	12.43	2.93	NA	2.26	3.01	3.8	NA	0.994
PC O-38:3	Se	21.29	13.22	26.76	6.48	3.41	14.53	0.001	4.87	3.35	12.51	0.001	0.918
PC O-38:3	SF	NA	NA	NA	2.02	0.44	6.21	NA	3.3	1.17	6.45	NA	0.841
PC O-38:3	Se/SF	NA	NA	NA	3.2	7.73	2.34	NA	1.48	2.86	1.94	NA	0.994
PC O-38:4	Se	10.08	8.01	11.46	11.19	8.64	14.52	0.158	11.75	9.31	14.15	0.07	0.918
PC O-38:4	SF	NA	NA	NA	2.52	1.22	4.39	NA	2.58	1.82	3.82	NA	0.914
PC O-38:4	Se/SF	NA	NA	NA	4.43	7.1	3.31	NA	4.55	5.11	3.71	NA	0.916

PC O-38:5	Se	7.33	6.36	11.41	13.63	9.81	17.63	0.001	15.3	10.42	21.88	<0.001	0.918
PC O-38:5	SF	NA	NA	NA	3.13	1.56	5.84	NA	3.19	2.09	5.19	NA	0.843
PC O-38:5	Se/SF	NA	NA	NA	4.36	6.31	3.02	NA	4.8	4.99	4.22	NA	0.916
PC O-40:4	Se	5.56	3.75	7.5	2.81	1.71	6.23	0.051	2.19	1.29	4.85	0.002	0.918
PC O-40:4	SF	NA	NA	NA	0.86	0.18	1.78	NA	1.64	0.63	2.43	NA	0.527
PC O-40:4	Se/SF	NA	NA	NA	3.27	9.56	3.49	NA	1.33	2.06	2	NA	0.592
PC O-40:5	Se	5.8	3.6	8.09	4.28	2.65	6.47	0.087	2.87	2.07	5.2	0.004	0.918
PC O-40:5	SF	NA	NA	NA	0.88	0.29	1.98	NA	1.88	0.95	3.06	NA	0.527
PC O-40:5	Se/SF	NA	NA	NA	4.89	9.2	3.26	NA	1.53	2.18	1.7	NA	0.403
PC O-40:6	Se	1.9	1.39	3.19	2.79	2.16	3.57	0.095	2.83	2.03	3.35	0.068	1
PC O-40:6	SF	NA	NA	NA	0.4	0.21	0.83	NA	0.79	0.42	1	NA	0.527
PC O-40:6	Se/SF	NA	NA	NA	7.03	10.32	4.3	NA	3.56	4.84	3.35	NA	0.873
PE 32:1	Se	0.24	0.21	0.35	0.3	0.25	0.4	0.126	0.33	0.24	0.51	0.013	0.378
PE 32:1	SF	0.05	0.03	0.15	0.1	0.06	0.11	0.502	0.07	0.05	0.13	0.737	0.963
PE 32:1	Se/SF	5.43	8.21	2.32	3.09	4.49	3.51	0.77	4.75	4.69	4	0.3	0.359
PE 34:1	Se	1.45	1	2.12	1.82	1.31	3.11	0.126	2.18	1.7	3.07	0.004	0.327
PE 34:1	SF	0.11	0.07	0.29	0.29	0.21	0.35	0.14	0.31	0.21	0.42	0.099	0.963
PE 34:1	Se/SF	13.73	14.62	7.35	6.19	6.32	8.8	0.029	6.99	8	7.28	0.001	0.359
PE 34:2	Se	2.13	1.6	3.11	3.16	1.95	3.92	0.126	3.24	2.54	5.8	0.003	0.327
PE 34:2	SF	0.12	0.08	0.35	0.37	0.24	0.47	0.125	0.36	0.22	0.47	0.093	0.963
PE 34:2	Se/SF	17.51	19.6	8.84	8.61	8.17	8.39	0.011	8.89	11.75	12.21	<0.001	0.5
PE 34:3	Se	0.36	0.31	0.51	0.31	0.29	0.5	0.233	0.41	0.28	0.56	0.977	0.327
PE 34:3	SF	0.05	0.03	0.1	0.11	0.08	0.15	0.125	0.09	0.05	0.14	0.255	0.963
PE 34:3	Se/SF	7.92	12.19	5.04	2.97	3.6	3.44	0.006	4.81	5.27	4.08	0.075	0.359

PE 36:1	Se	0.51	0.32	0.63	0.55	0.39	0.73	0.241	0.65	0.55	1.09	0.002	0.327
PE 36:1	SF	0.09	0.06	0.23	0.1	0.08	0.16	0.979	0.11	0.09	0.15	0.998	0.963
PE 36:1	Se/SF	5.82	5.45	2.77	5.28	4.77	4.63	0.236	6.06	6.34	7.31	0.001	0.308
PE 36:2	Se	1.73	1.26	2.46	2.8	1.72	3.83	0.042	2.94	2.29	4.82	<0.001	0.327
PE 36:2	SF	0.24	0.18	0.78	0.29	0.18	0.45	0.979	0.31	0.2	0.43	0.998	0.963
PE 36:2	Se/SF	7.24	7.14	3.17	9.8	9.35	8.42	0.136	9.37	11.29	11.21	0.008	0.359
PE 36:3	Se	0.61	0.43	0.82	0.78	0.5	1.04	0.276	0.83	0.63	1.26	0.013	0.327
PE 36:3	SF	0.1	0.05	0.26	0.11	0.09	0.15	0.979	0.11	0.08	0.14	1	0.963
PE 36:3	Se/SF	6.09	8.63	3.17	6.99	5.57	7.02	0.296	7.22	7.62	9	0.01	0.359
PE 36:4	Se	0.54	0.4	0.9	1.03	0.62	1.43	0.013	1.14	0.76	1.75	<0.001	0.327
PE 36:4	SF	0.15	0.07	0.26	0.12	0.08	0.17	0.979	0.13	0.08	0.17	0.998	0.963
PE 36:4	Se/SF	3.66	6.03	3.5	8.83	7.27	8.2	0.011	8.44	9.65	10.26	<0.001	0.359
PE 38:3	Se	0.41	0.29	0.48	0.6	0.47	0.9	0.005	0.8	0.54	1.05	<0.001	0.327
PE 38:3	SF	0.09	0.06	0.19	0.14	0.11	0.17	0.979	0.12	0.08	0.16	0.998	0.963
PE 38:3	Se/SF	4.67	4.58	2.61	4.46	4.38	5.35	0.025	6.82	6.71	6.56	<0.001	0.359
PE 38:4	Se	2.25	1.28	3.04	4.62	2.4	6.69	0.001	4.69	3.26	7.87	<0.001	0.327
PE 38:4	SF	0.57	0.28	1.1	0.51	0.34	0.72	0.979	0.59	0.32	0.88	0.998	0.963
PE 38:4	Se/SF	3.98	4.64	2.78	9.11	7.07	9.31	0.241	7.98	10.34	8.9	0.056	0.359
PE 38:5	Se	0.81	0.57	1.14	1.4	0.79	1.84	0.005	1.54	0.93	2.62	<0.001	0.327
PE 38:5	SF	0.18	0.12	0.37	0.19	0.15	0.3	0.979	0.21	0.14	0.35	0.998	0.963
PE 38:5	Se/SF	4.58	4.83	3.08	7.21	5.42	6.05	0.006	7.5	6.64	7.46	<0.001	0.359
PE 38:6	Se	0.81	0.47	1.52	2	0.96	2.75	0.005	2.18	1.37	4.42	<0.001	0.327
PE 38:6	SF	0.21	0.14	0.41	0.21	0.13	0.35	0.979	0.27	0.16	0.39	0.998	0.963
PE 38:6	Se/SF	3.91	3.28	3.66	9.35	7.42	7.82	0.006	7.99	8.39	11.48	<0.001	0.359

PE P-16:0/18:1	Se	1.85	1.64	2.2	2.14	1.86	2.66	0.039	2.6	1.94	3.1	0.001	0.294
PE P-16:0/18:1	SF	0.37	0.24	0.52	0.89	0.64	1.23	0.008	0.77	0.63	0.95	0.011	0.46
PE P-16:0/18:1	Se/SF	4.99	6.76	4.27	2.4	2.89	2.16	0.203	3.38	3.06	3.25	0.024	0.967
PE P-16:0/18:2	Se	2.67	2.17	3.68	3.85	3.04	4.94	0.003	4.93	4.14	6.3	<0.001	0.146
PE P-16:0/18:2	SF	0.65	0.38	0.84	1	0.78	1.51	0.013	1.03	0.81	1.36	0.045	0.89
PE P-16:0/18:2	Se/SF	4.11	5.76	4.39	3.84	3.9	3.27	0.751	4.8	5.14	4.65	0.598	0.967
PE P-16:0/20:3	Se	1.25	1.08	1.4	1.67	1.5	2.03	<0.001	1.88	1.65	2.46	<0.001	0.215
PE P-16:0/20:3	SF	0.24	0.15	0.46	0.69	0.44	0.98	0.013	0.55	0.36	0.72	0.045	0.46
PE P-16:0/20:3	Se/SF	5.29	7.12	3.03	2.42	3.37	2.07	0.016	3.43	4.58	3.42	0.001	0.967
PE P-16:0/20:4	Se	3.73	2.96	6.46	8.42	6.16	10.03	<0.001	10.54	5.56	15.59	<0.001	0.278
PE P-16:0/20:4	SF	1.33	0.78	1.61	1.95	1.5	2.28	0.013	2.17	1.5	2.9	0.011	0.46
PE P-16:0/20:4	Se/SF	2.81	3.8	4.01	4.31	4.09	4.4	0.751	4.86	3.7	5.37	0.748	0.967
PE P-16:0/20:5	Se	1.7	1.38	2.06	2.12	1.79	2.54	0.018	2.46	1.76	3.24	0.001	0.319
PE P-16:0/20:5	SF	0.28	0.19	0.39	0.88	0.57	1.17	0.002	0.77	0.48	1.08	0.008	0.46
PE P-16:0/20:5	Se/SF	6	7.1	5.23	2.41	3.14	2.18	0.205	3.2	3.66	3.01	0.019	0.967
PE P-16:0/22:4	Se	1.16	1.09	1.37	1.51	1.34	1.7	<0.001	1.47	1.35	2.11	<0.001	0.507
PE P-16:0/22:4	SF	0.36	0.28	0.76	0.7	0.59	1.09	0.028	0.62	0.54	0.81	0.077	0.46
PE P-16:0/22:4	Se/SF	3.24	3.92	1.81	2.15	2.26	1.56	0.042	2.37	2.49	2.59	0.017	0.967
PE P-16:0/22:5	Se	1.55	1.36	1.72	2.72	2.17	3.35	<0.001	3.12	2.03	4.74	<0.001	0.371
PE P-16:0/22:5	SF	0.36	0.28	0.85	0.83	0.72	1.17	0.013	0.8	0.64	0.97	0.045	0.46
PE P-16:0/22:5	Se/SF	4.33	4.87	2.02	3.26	3.03	2.86	0.247	3.9	3.17	4.86	0.035	0.967
PE P-16:0/22:6	Se	2.33	1.98	3.04	4.06	3.12	7.06	<0.001	5.93	3.8	7.78	<0.001	0.25
PE P-16:0/22:6	SF	0.52	0.31	1.17	1.22	0.86	1.58	0.013	1.39	0.94	1.79	0.011	0.593
PE P-16:0/22:6	Se/SF	4.52	6.38	2.6	3.34	3.62	4.48	0.275	4.28	4.06	4.34	0.02	0.967

PE P-18:0/18:1	Se	1.55	1.2	1.88	2.02	1.48	2.68	0.014	2.55	2.03	2.99	<0.001	0.128
PE P-18:0/18:1	SF	0.26	0.22	0.7	0.62	0.46	0.84	0.082	0.66	0.46	0.79	0.088	0.963
PE P-18:0/18:1	Se/SF	5.94	5.42	2.68	3.24	3.24	3.19	0.018	3.84	4.45	3.77	0.002	0.744
PE P-18:0/18:2	Se	3.08	2.46	4.35	5.6	3.5	6.37	0.003	6.21	4.51	8.16	<0.001	0.128
PE P-18:0/18:2	SF	0.42	0.32	0.77	0.88	0.71	1.48	0.022	1.24	0.89	1.41	0.011	0.773
PE P-18:0/18:2	Se/SF	7.26	7.59	5.63	6.37	4.95	4.29	0.839	5.01	5.04	5.79	0.462	0.849
PE P-18:0/20:3	Se	0.93	0.8	1.27	1.47	0.93	1.76	0.012	1.5	1.37	2.51	<0.001	0.128
PE P-18:0/20:3	SF	0.23	0.14	0.53	0.54	0.41	0.75	0.082	0.47	0.33	0.56	0.159	0.773
PE P-18:0/20:3	Se/SF	4	5.76	2.38	2.71	2.24	2.34	0.008	3.21	4.19	4.44	<0.001	0.744
PE P-18:0/20:4	Se	4.76	3.77	8.23	9.71	7.97	14.69	<0.001	15.99	8.49	20.35	<0.001	0.128
PE P-18:0/20:4	SF	0.82	0.48	1.69	2.02	1.45	2.74	0.022	2.23	1.46	2.76	0.002	0.773
PE P-18:0/20:4	Se/SF	5.79	7.79	4.87	4.8	5.5	5.36	0.164	7.16	5.83	7.36	0.166	0.925
PE P-18:0/20:5	Se	1.55	1.18	2.05	1.99	1.53	2.57	0.022	2.21	1.79	3.57	<0.001	0.128
PE P-18:0/20:5	SF	0.31	0.15	0.49	0.71	0.51	1.05	0.022	0.76	0.45	0.94	0.022	0.912
PE P-18:0/20:5	Se/SF	4.94	7.98	4.17	2.82	2.99	2.45	0.043	2.9	4.01	3.79	0.007	0.793
PE P-18:0/22:5	Se	0.9	0.77	1.06	1.3	1.04	1.86	<0.001	1.7	1.27	2.08	<0.001	0.128
PE P-18:0/22:5	SF	0.23	0.13	0.55	0.51	0.33	0.68	0.082	0.46	0.34	0.54	0.136	0.773
PE P-18:0/22:5	Se/SF	3.84	6.09	1.91	2.56	3.13	2.73	0.003	3.69	3.72	3.83	<0.001	0.744
PE P-18:0/22:6	Se	1.38	1.12	2.31	3.2	2.37	3.62	<0.001	3.7	2.96	4.93	<0.001	0.128
PE P-18:0/22:6	SF	0.35	0.25	0.75	0.77	0.54	1.04	0.082	0.91	0.61	1.1	0.049	0.773
PE P-18:0/22:6	Se/SF	3.92	4.46	3.07	4.13	4.4	3.47	0.066	4.07	4.88	4.47	0.039	0.757
PE P-18:1/16:0	Se	1.16	0.98	1.31	1.36	1.23	1.67	0.003	1.49	1.26	1.63	<0.001	0.507
PE P-18:1/16:0	SF	0.29	0.14	0.39	0.57	0.35	0.9	0.013	0.47	0.31	0.62	0.045	0.46
PE P-18:1/16:0	Se/SF	3.98	7.06	3.32	2.36	3.5	1.85	0.016	3.2	4.07	2.61	0.001	0.967

PE P-18:1/16:1	Se	0.97	0.88	1.11	1.19	1.01	1.26	0.003	1.23	1.13	1.37	<0.001	0.319
PE P-18:1/16:1	SF	0.26	0.12	0.46	0.59	0.26	0.91	0.013	0.35	0.25	0.61	0.069	0.46
PE P-18:1/16:1	Se/SF	3.72	7.31	2.42	2.01	3.85	1.39	0.016	3.5	4.56	2.24	<0.001	0.967
PE P-18:1/18:0	Se	0.96	0.85	1.03	1.04	0.9	1.17	0.022	1.08	0.91	1.18	0.026	0.926
PE P-18:1/18:0	SF	0.23	0.11	0.37	0.54	0.26	0.78	0.013	0.36	0.22	0.58	0.054	0.46
PE P-18:1/18:0	Se/SF	4.25	7.75	2.76	1.92	3.52	1.5	0.136	2.97	4.18	2.02	0.033	0.967
PE P-18:1/18:1	Se	1.61	1.38	1.82	2.08	1.67	2.43	0.001	2.52	2.03	3.07	<0.001	0.146
PE P-18:1/18:1	SF	0.32	0.2	0.52	0.86	0.55	1.05	0.013	0.68	0.54	0.87	0.011	0.46
PE P-18:1/18:1	Se/SF	5.02	6.99	3.5	2.42	3.02	2.32	0.058	3.72	3.76	3.54	0.004	0.967
PE P-18:1/18:2	Se	1.91	1.67	2.53	3.06	2.15	3.43	0.001	3.96	2.92	5.14	<0.001	0.146
PE P-18:1/18:2	SF	0.48	0.33	0.78	0.9	0.68	1.39	0.013	0.84	0.61	1.03	0.042	0.509
PE P-18:1/18:2	Se/SF	3.94	5.12	3.25	3.39	3.15	2.47	0.247	4.71	4.77	4.97	0.04	0.967
PE P-18:1/20:3	Se	0.78	0.64	0.82	0.96	0.72	1.07	0.006	1.1	0.83	1.41	<0.001	0.128
PE P-18:1/20:3	SF	0.2	0.14	0.48	0.43	0.26	0.61	0.157	0.38	0.25	0.49	0.268	0.773
PE P-18:1/20:3	Se/SF	3.95	4.48	1.71	2.24	2.8	1.75	0.15	2.89	3.3	2.87	0.002	0.516
PE P-18:1/20:4	Se	2.89	2.11	4.22	5.33	4.36	7.04	<0.001	7.88	4.99	9.9	<0.001	0.128
PE P-18:1/20:4	SF	0.87	0.52	1.62	1.16	0.93	1.63	0.318	1.39	1	1.81	0.237	0.773
PE P-18:1/20:4	Se/SF	3.34	4.05	2.6	4.61	4.67	4.31	0.19	5.66	5.01	5.47	0.244	0.793
PE P-18:1/20:5	Se	1.07	0.94	1.48	1.32	0.97	1.44	0.227	1.44	1.17	2.18	0.005	0.128
PE P-18:1/20:5	SF	0.29	0.16	0.55	0.63	0.37	0.88	0.082	0.55	0.33	0.86	0.136	0.773
PE P-18:1/20:5	Se/SF	3.73	6.02	2.7	2.08	2.65	1.64	0.1	2.63	3.56	2.52	0.006	0.744
PE P-18:1/22:6	Se	0.95	0.88	1.51	1.8	1.53	2.52	<0.001	2.4	1.51	3.2	<0.001	0.128
PE P-18:1/22:6	SF	0.38	0.19	0.89	0.59	0.37	0.83	0.318	0.53	0.46	0.66	0.268	0.773
PE P-18:1/22:6	Se/SF	2.54	4.55	1.69	3.04	4.16	3.05	0.003	4.57	3.26	4.85	<0.001	0.757

SM 32:1;O2	Se	7.61	6.09	9.53	10.07	8.15	11.98	0.028	8.36	7.3	13.99	0.04	0.758
SM 32:1;O2	SF	1.39	0.92	1.59	2.23	1.55	4.32	0.026	3.62	2.06	5.18	0.001	0.991
SM 32:1;O2	Se/SF	5.46	6.65	5.97	4.52	5.25	2.78	0.402	2.31	3.54	2.7	0.316	0.983
SM 33:1;O2	Se	4.48	3.79	6.03	5.11	4.53	6.71	0.148	5.65	4.2	6.93	0.073	0.758
SM 33:1;O2	SF	0.68	0.49	0.98	1.21	0.48	2.51	0.192	1.64	1.26	2.33	0.001	0.991
SM 33:1;O2	Se/SF	6.61	7.66	6.17	4.24	9.49	2.68	0.948	3.45	3.35	2.97	0.001	0.983
SM 34:0;O2	Se	3.75	3.04	5.62	4.76	3.73	6.88	0.148	5.36	4.25	7.2	0.045	0.758
SM 34:0;O2	SF	0.54	0.45	0.86	1.68	0.52	2.88	0.045	1.62	0.89	2.42	0.002	0.991
SM 34:0;O2	Se/SF	6.94	6.8	6.53	2.84	7.13	2.39	0.982	3.31	4.75	2.98	0.567	0.983
SM 34:1;O2	Se	74.99	63.49	98.94	98.18	79.58	127.2	0.028	104.75	85.12	137.11	0.012	0.758
SM 34:1;O2	SF	17.74	14.04	21.81	31.26	13.95	53.38	0.083	32.57	24.86	46.59	0.015	0.991
SM 34:1;O2	Se/SF	4.23	4.52	4.54	3.14	5.7	2.38	0.294	3.22	3.42	2.94	0.227	0.983
SM 34:2;O2	Se	13.82	11.56	18.98	18.99	15.81	25.62	0.028	22.44	17.84	29.88	0.002	0.758
SM 34:2;O2	SF	2.18	1.85	3.23	7.73	4.11	11.64	0.002	8.07	4.57	9.37	<0.001	0.991
SM 34:2;O2	Se/SF	6.33	6.25	5.88	2.46	3.85	2.2	0.025	2.78	3.9	3.19	0.023	0.983
SM 36:1;O2	Se	16.3	12.23	19.6	18.82	14.83	25.78	0.127	21.35	16.37	27.44	0.02	0.758
SM 36:1;O2	SF	2.22	1.67	3.75	3.75	1.96	7.44	0.083	3.94	3.17	7.14	0.015	0.991
SM 36:1;O2	Se/SF	7.34	7.33	5.23	5.01	7.57	3.46	0.294	5.42	5.16	3.84	0.223	0.983
SM 36:2;O2	Se	10.26	8.1	12.88	12.68	10.27	17.02	0.028	12.63	10.82	17.2	0.014	0.758
SM 36:2;O2	SF	0.99	0.92	1.76	2.8	0.93	5	0.024	3.28	2.1	4.15	<0.001	0.991
SM 36:2;O2	Se/SF	10.36	8.8	7.3	4.52	11.06	3.4	0.948	3.85	5.15	4.14	0.826	0.983
SM 38:2;O2	Se	5.63	4.28	8.14	6.91	4.38	8.19	0.351	7.05	5.32	9.25	0.04	0.758
SM 38:2;O2	SF	0.55	0.37	1.06	1.75	0.77	2.51	0.019	1.38	0.88	2.3	0.002	0.991
SM 38:2;O2	Se/SF	10.26	11.5	7.71	3.94	5.68	3.27	0.948	5.11	6.02	4.03	0.461	0.983

SM 40:1;O2	Se	18.34	15.67	23.67	26.38	18.82	31.24	0.028	22.45	19.49	27.03	0.042	0.758
SM 40:1;O2	SF	0.99	0.3	1.89	4.84	2.57	8.95	0.001	5.35	3.19	8.56	<0.001	0.991
SM 40:1;O2	Se/SF	18.52	51.61	12.54	5.45	7.33	3.49	0.446	4.2	6.1	3.16	0.414	0.983
SM 40:2;O2	Se	23.18	18.61	30.22	26.59	20.83	32.19	0.148	26.39	22.86	31.94	0.061	0.758
SM 40:2;O2	SF	1.85	1.05	2.65	5.28	3.03	11.06	0.002	7.09	4.35	8.32	<0.001	0.991
SM 40:2;O2	Se/SF	12.53	17.65	11.43	5.03	6.88	2.91	0.069	3.72	5.26	3.84	0.054	0.983
SM 42:1;O2	Se	13.47	11.06	16	16.36	13.49	20.6	0.095	15.1	13.41	16.71	0.061	0.758
SM 42:1;O2	SF	1.43	0.74	2.18	3.19	1.62	7.65	0.011	4.25	2.23	5.44	<0.001	0.991
SM 42:1;O2	Se/SF	9.44	15.01	7.35	5.14	8.33	2.69	0.402	3.55	6	3.07	0.348	0.983
SM 42:2;O2	Se	58.6	47.97	73.13	61.78	52.13	75.49	0.296	66.39	58.49	88.28	0.079	0.758
SM 42:2;O2	SF	5.89	3.75	9.46	20.91	8.86	32.51	0.003	18.28	13.34	28.03	<0.001	0.991
SM 42:2;O2	Se/SF	9.95	12.79	7.73	2.95	5.89	2.32	0.01	3.63	4.38	3.15	0.003	0.983
SM 42:3;O2	Se	23.98	20.44	28.03	25.47	20.66	32.29	0.436	30.53	22.49	34.46	0.107	0.758
SM 42:3;O2	SF	3.88	2.55	4.96	11.13	5.6	14.98	0.014	9.69	6.11	13.44	0.002	0.991
SM 42:3;O2	Se/SF	6.19	8.01	5.65	2.29	3.69	2.16	0.034	3.15	3.68	2.56	0.032	0.983

Supplementary Table 2: FDR-adj. p-values values of Wilcoxon´s rank sum test for serum and for SF data, and of custom-made test for Se/SF ratio data; adj., adjusted; C, control; Cer, ceramide; eOA, early stage osteoarthritis; HexCer, hexosylceramide; lOA, late stage osteoarthritis; LPC, lysophosphatidylcholine; PC, phosphatidylcholine; PC O, ether-linked phosphatidylcholine; PE, phosphatidylethanolamine; PE P, phosphatidylethanolamine based plasmalogen; Q, quartile; Se, serum; SF, synovial fluid; SM, sphingomyelin.
